# αvβ1 integrin is enriched in extracellular vesicles of metastatic breast cancer cells: A mechanism mediated by galectin‐3

**DOI:** 10.1002/jev2.12234

**Published:** 2022-08-03

**Authors:** Daniel Xin Zhang, Xuan T. T. Dang, Luyen Tien Vu, Claudine Ming Hui Lim, Eric Yew Meng Yeo, Brenda Wan Shing Lam, Sai Mun Leong, Noorjehan Omar, Thomas Choudary Putti, Yu Chen Yeh, Victor Ma, Jia‐Yuan Luo, William C. Cho, Gang Chen, Victor Kwan Min Lee, Andrew Grimson, Minh T. N. Le

**Affiliations:** ^1^ Department of Pharmacology and Institute for Digital Medicine Yong Loo Lin School of Medicine National University of Singapore Queenstown Singapore; ^2^ Department of Surgery Cancer Program, Immunology Program and Nanomedicine Translational Research Program Yong Loo Lin School of Medicine National University of Singapore Queenstown Singapore; ^3^ Department of Biomedical Sciences Jocky Club College of Veterinary Medicine and Life Sciences City University of Hong Kong Kowloon Hong Kong SAR; ^4^ Department of Molecular Biology and Genetics Cornell University Ithaca New York USA; ^5^ Department of Pathology Yong Loo Lin School of Medicine National University of Singapore Queenstown Singapore; ^6^ Department of Clinical Oncology Queen Elizabeth Hospital Kowloon Hong Kong SAR; ^7^ Department of Pathology The First Affiliated Hospital of Guangxi Medical University Nanning China

**Keywords:** cancer, galectin, integrin, metastasis, mechanism, microenvironment, sorting

## Abstract

Breast cancer cells release a large quantity of biocargo‐bearing extracellular vesicles (EVs), which mediate intercellular communication within the tumour microenvironment and promote metastasis. To identify EV‐bound proteins related to metastasis, we used mass spectrometry to profile EVs from highly and poorly metastatic breast cancer lines of human and mouse origins. Comparative mass spectrometry indicated that integrins, including αv and β1 subunits, are preferentially enriched in EVs of highly metastatic origin over those of poorly metastatic origin. These results are consistent with our histopathological findings, which show that integrin αv is associated with disease progression in breast cancer patients. Integrin αv colocalizes with the multivesicular‐body marker CD63 at a higher frequency in the tumour and is enriched in circulating EVs of breast cancer patients at late stages when compared with circulating EVs from early‐stage patients. With a magnetic bead‐based flow cytometry assay, we confirmed that integrins αv and β1 are enriched in the CD63^+^ subsets of EVs from both human and mouse highly metastatic cells. By analysing the level of integrin αv on circulating EVs, this assay could predict the metastatic potential of a xenografted mouse model. To explore the export mechanism of integrins into EVs, we performed immunoprecipitation mass spectrometry and identified members of the galectin family as potential shuttlers of integrin αvβ1 into EVs. In particular, knockdown of galectin‐3, but not galectin‐1, causes a reduction in the levels of cell surface integrins β1 and αv, and decreases the colocalization of these integrins with CD63. Importantly, knockdown of galectin‐3 leads to a decrease of integrin αvβ1 export into the EVs concomitant with a decrease in the metastatic potential of breast cancer cells. Moreover, inhibition of the integrin αvβ1 complex leads to a reduction in the binding of EVs to fibronectin, suggesting that integrin αvβ1 is important for EV retention in the extracellular matrix. EVs retained in the extracellular matrix are taken up by fibroblasts, which differentiate into cancer associated fibroblasts. In summary, our data indicate an important link between EV‐bound integrin αvβ1 with breast cancer metastasis and provide additional insights into the export of integrin αvβ1 into EVs in the context of metastasis.

## 1 INTRODUCTION

Breast cancer is regarded as the most common cancer in women globally, accounting for one fourth of cancer incidences and one sixth of cancer‐related mortalities among women (Sung et al., 2021). Although early‐stage, non‐metastatic breast cancer is often curable with locoregional and systemic therapies, advanced‐stage, metastatic breast cancer is considered incurable with current treatment options and has poor prognosis (Waks & Winer, 2019). Therefore, it is critical to identify patients with higher metastatic risks at an early stage and to elucidate the underlying mechanisms of breast cancer metastasis and progression.

Extracellular vesicles (EVs) are a burgeoning research field in cell biology. EVs are membrane‐bound vesicles secreted by various cell types and can derive from both the release of intraluminal vesicles by fusion of multivesicular bodies with the plasma membrane and the outward budding and blebbing of the plasma membrane (Van Niel et al., 2018). In the tumour microenvironment, EVs transport many bioactive cargoes including proteins and RNAs, mediating a variety of interactions with cells and non‐cell components within tumours, and can potentiate malignant transformation and metastatic phenotypes (Zhang et al., 2021). These findings have inspired a number of basic studies focusing on the underlying mechanisms of cancer development and progression (Zhang et al., 2021) as well as translational research investigating the utility of EVs as cancer‐associated biomarkers (Shah et al., 2018, Xu et al., 2018) and delivery vehicles of anticancer agents (Möller & Lobb, 2020, Zhang et al., 2019).

Among different EV‐bound molecules, integrins, a family of transmembrane proteins composed of diverse α and β subunits that have multifaceted functions in cell adhesion and cell signalling (Cooper & Giancotti, 2019), are known to play a crucial role in metastasis development (Mo et al., 2020, Paolillo & Schinelli, 2017). There is now considerable interest in EV‐bound integrins in cancer research, initiated by a landmark study in 2015, which demonstrated the pivotal role of EV‐bound integrins in organotropic metastasis (Hoshino et al., 2015). Primary tumour cells secrete organotropic EVs bearing different heterodimeric integrins αvβ5, α6β1 and α6β4, which are preferentially taken up by resident cells in distant metastatic sites such as fibroblasts and epithelial cells in the lung and Kupffer cells in the liver. Preferential uptake of such tumour cell‐derived EVs directs resident cells to activate Src phosphorylation and upregulate gene expression of the pro‐inflammatory S100 family, which prepares a favourable microenvironment for new colony formation and growth of metastatic cancer cells. In addition, targeting these EV‐bound integrins inhibits EV uptake and reduces distant metastasis. Colorectal cancer cells are found to elicit integrin beta‐like 1‐enriched EVs to target fibroblasts for premetastatic niche formation (Ji et al., 2020), while EVs from epithelial ovarian cancer cells bear abundant integrin α5β1 complexed with asparaginyl endopeptidase to target human peritoneal mesothelial cells for peritoneal metastasis (Li et al., 2020). Additional studies have shown important roles of different EV‐bound integrin heterodimers to promote angiogenesis (Krishn et al., 2020) and cell migration (Fedele et al., 2015), to induce neuroendocrine differentiation (Quaglia et al., 2020), and to modulate the homing of lymphocytes in intestinal tissues (Shimaoka et al., 2019). More importantly, a recent large‐scale proteomic study, which compared EV protein cargo from sixty diverse human cancer cell lines, including breast cancer lines, demonstrated that levels of integrins αv, α6, and β1 correlate with cancer progression amongst a broad variety of epithelial cancer cell‐derived EVs (Hurwitz & Meckes, 2019).

Previously, we have demonstrated the horizontal transfer of metastatic traits dependant on EV‐bound microRNA‐200 (miR‐200), using a series of isogenic mouse breast cancer cell lines (Le et al., 2014). In addition, we have reported the pivotal role of miR‐125b enclosed within breast cancer cell‐derived EVs in activating cancer‐associated fibroblasts (CAFs) in the tumour microenvironment (Vu et al., 2019). In this work, we investigated the associations of EV‐bound integrins with breast cancer metastasis as well as the utility and export mechanisms of integrins in EVs. Specifically, to identify the differentially expressed integrins associated with metastasis, we adopted a two‐step purification method to obtain ultrapure EVs and performed comparative mass spectrometry to profile EVs from human and mouse breast cancer lines of different metastatic capabilities. Then, we focused on the integrin candidates that were consistent in both human and mouse cell lines and validated the overexpression of integrins αv and β1 in EVs of highly metastatic origin using a magnetic bead‐based flow cytometry assay. Applying this assay to integrin αv in circulating EVs, we were able to evaluate metastatic potential using a xenografted mouse model. The results from these experiments, which were also consistent with our histopathological data, indicate that integrin αv in EVs is associated with progression in breast cancer patients. Furthermore, we demonstrated that the export of integrin αvβ1 into EVs is, in part, regulated by galectin‐3 and that integrin αvβ1 is important for the fibronectin‐based retention of EVs that promotes fibroblast differentiation. This work highlights the association of integrin αvβ1 with breast cancer metastasis, and suggests the potential of EV‐bound integrin αvβ1 as a novel prognostic and therapeutic target in breast cancer metastasis.

## 2 RESULTS

### 2.1 Purification of extracellular vesicles from breast cancer cells

**FIGURE 1:**
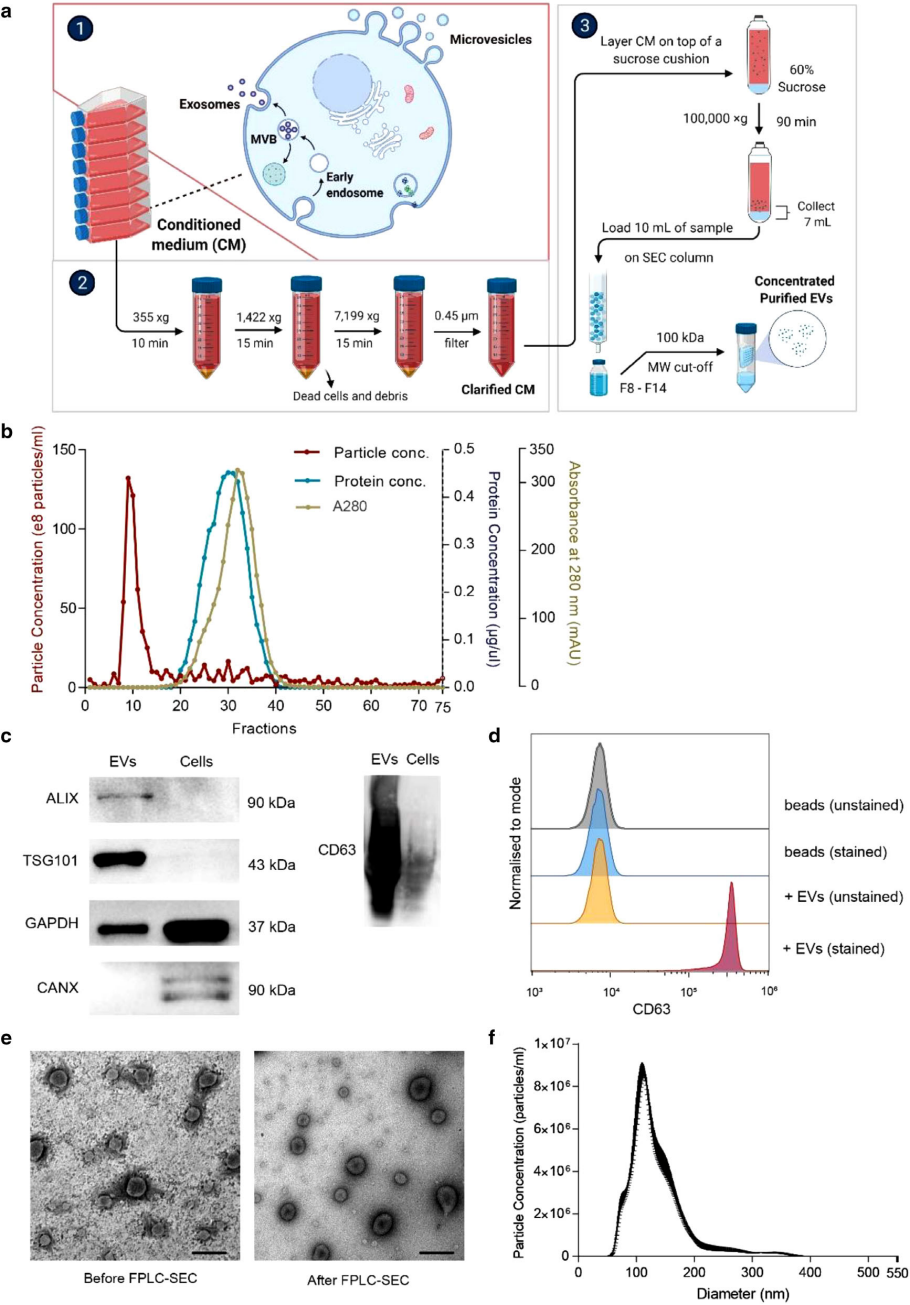
Isolation and characterization of EVs from breast cancer cells. (a) Schema of isolation strategy to obtain breast cancer cell‐derived EVs from conditioned medium using a two‐step EV isolation regimen combining flotation ultracentrifugation and fast protein size‐exclusion liquid chromatography (FPLC‐SEC). Purified EVs were combined from SEC fraction 8 to 14 and concentrated using a filter unit with 100‐kDa cut‐off. (b) Separation of EVs from vesicle‐free proteins using FPLC‐SEC, indicated by the concentrations of EVs as determined using nanoparticle tracking analysis, and the concentration of free proteins, determined using BCA assay and absorbance at 280 nm in each fraction of the FPLC‐SEC. (c) Western blot analysis of proteins in CA1a cells and purified EVs. (d) Flow cytometry analysis of CD63 in purified EVs. (e) Representative images of EVs before and after FPLC‐SEC (fraction 8–14 were combined), obtained by transmission electron microscopy. Scale bars: 0.5 μm. (f) Size distribution of the isolated EVs, determined by nanoparticle tracking analysis (100x diluted)

Cancer cells naturally secrete biomolecule‐carrying EVs into the extracellular space to mediate metastasis and other related biological processes (Becker et al., 2016). To obtain ultrapure EVs devoid of protein contaminants, we applied a two‐step purification regimen combining flotation ultracentrifugation and fast protein size‐exclusion liquid chromatography (FPLC‐SEC) (Figure 1a). We cultured breast cancer cells with EV‐depleted medium and harvested the conditioned medium (CM), which we subjected to serial centrifugation and 0.45 μm filtering to remove dead cells and debris. Clarified CM went through flotation ultracentrifugation with a 60% sucrose cushion and then FPLC‐SEC. FPLC‐SEC fractions were collected individually for analysis, and we found that EVs were enriched predominantly in fractions 8–14, indicated by particle peaks detected using nanoparticle tracking analysis, while vesicle‐free proteins were detected in fractions 19–41, as determined by bicinchoninic acid (BCA) assay and absorbance at 280 nm (Figure 1b). The high purity of isolated EVs can be deduced from the distinctly separated peaks for EVs and vesicle‐free proteins, respectively. After FPLC‐SEC, we concentrated EVs using a centrifugal concentrator for characterisation. Using western blots, we observed increased levels of EV markers ALIX, TSG101, and CD63, and reduced level of cellular markers GAPDH in the isolated EVs as compared to the parental cells (Figure 1c and Figure S1A). Moreover, the absence of the endoplasmic reticulum marker CANX in the purified EVs indicates that the EVs were free from cellular contamination (Figure 1c and Figure S1A). Flow cytometry analysis further confirmed the enrichment of CD63 on the EV surface (Figure 1d). EVs collected in fractions 8–14 demonstrated a typical cup‐shape and double‐membrane morphology when examined by transmission electron microscopy, indicating that the purified EVs were intact (Figure 1e). Nanoparticle tracking analysis revealed that the diameters of isolated EVs ranged from 50 to 380 nm, with ∼ 115 nm particles the most prominent, as expected for EVs (Figure 1f). Collectively, these data show that our two‐step purification method can isolate highly pure and intact EVs for further analysis.

### 2.2 Identification of integrins in metastatic breast cancer‐derived EVs

**FIGURE 2:**
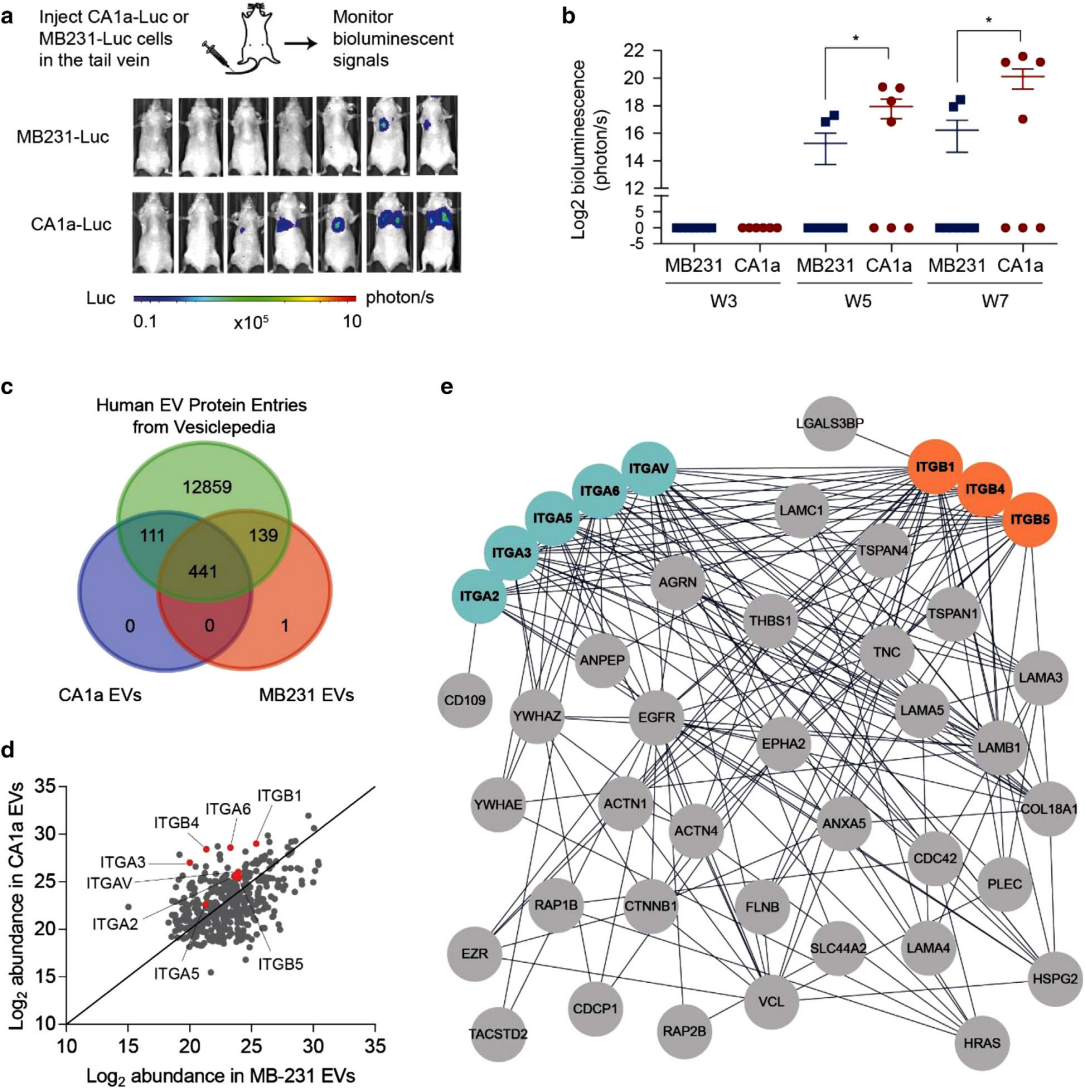
Proteomic profiling of human breast cancer cell‐derived EVs. (a) Injection of 5×10^6^ human breast cancer CA1a‐luc cells and 5×10^6^ MB‐231‐luc cells in the tail vein of nude mice resulted in lung metastasis that can be monitored by bioluminescent imaging. Shown are the bioluminescent images of the mice at 7 weeks. (b) Log_2_ bioluminescent signal (photon/s) in the lung area in week 3, 5 and 7. (c) Venn diagram showing overlaps of identified proteins in CA1a EVs and MB‐231 EVs from comparative proteomic profiling with known human EV protein entries from Vesiclepedia. (d) Scatter plot comparing the abundance of proteins identified in CA1a and MB‐231 EVs. (e) Protein‐protein interaction network analysis of proteins enriched in CA1a EVs compared to MB‐231 EVs. Student's t‐test *P ≤ 0.05

To examine EV proteins associated with metastasis, we compared EVs from two human breast cancer cell lines, MCF10CA1a (CA1a) and MDA‐MB‐231 (MB‐231), which have similar rates of proliferation and similar EV production. CA1a cells are the most metastatic in a series of cell lines derived from MCF10A cells (Santner et al., 2001). Mice xenografted with these cells have been used as a reliable metastatic model (Jin et al., 2009, Lei et al., 2014, Tian et al., 2003). MDA‐MB‐231 cells were derived originally from a breast cancer patient who suffered from metastasis (Cailleau et al., 1974). When inoculated into immunodeficient mice, MB‐231 cells are poorly metastatic although sub‐clones of these cells have been derived with enhanced metastatic potential (Kang et al., 2003). We have shown previously that CA1a cells have a higher metastatic potential than the original MDA‐MB‐231 cells (Le et al., 2014). To confirm the difference in metastatic capabilities of two human breast cancer cells available in our lab, we transduced CA1a cells and MB‐231 cells with a lentiviral plasmid expressing luciferase. CA1a‐luciferase (CA1a‐luc) and MB‐231‐luciferase (MB‐231‐luc) cells of equal counts were injected intravenously into nude mice via the tail vein, to generate metastatic models suitable for monitoring by bioluminescent imaging (Figure 2a). After 7 weeks, four out of seven nude mice injected with CA1‐luc cells showed bioluminescence signal in the lungs while only two out of seven mice injected with MB‐231‐luc cells showed sustained colonization in the lungs (Figure 2a‐b). The average bioluminescent signal in the lung of CA1a‐cell‐injected mice was significantly higher than in MB‐231‐cell‐injected mice. This demonstrated that CA1a cells have higher metastatic potential than MB‐231 cells in our experimental setting. In addition, we also selected a pair of isogenic mouse breast cancer cells of different metastatic capabilities: highly metastatic 4T1 cells and poorly metastatic 4TO7 cells (Le et al., 2014, Vu et al., 2019). This strategy was designed to allow us to identify proteins enriched in EVs derived from highly metastatic cells from both humans and mice.

To identify the EV‐bound integrins associated with breast cancer metastasis, we isolated EVs from breast cancer cells with different metastatic potentials and profiled the cancer‐derived EVs using comparative mass spectrometry. We identified 552 and 581 proteins in EVs derived from the human CA1a and MB‐231 cells, respectively, and, 634 and 568 proteins in the EVs derived from the mouse 4T1 EVs and 4TO7 cells, when requiring at least two unique peptides identified per protein (Figure 2c and Figure S1B). CA1a EVs and MB‐231 EVs had an overlap of 441 proteins while 4T1 EVs and 4TO7 EVs presented an overlap of 551 proteins (Figure 2c and Figure S1B). Gene ontology (GO) analysis of the overlapped protein entries pointed to ‘cell‐cell adhesion’, ‘cell adhesion’, and ‘signal transduction’ in biological processes, which are also broadly recognized functions of integrins (Table S1 and S2). Top‐ranked common GO entries in cellular components included ‘extracellular exosome’, ‘membrane’ and ‘extracellular space’, confirming the EV origin of the profiled proteins (Table S1 and S2). In terms of molecular functions in GO analysis, many protein binding‐related entries are prominent, suggesting potential active protein‐binding interactions among the EV proteins. Kyoto Encyclopedia of Genes and Genomes (KEGG) pathway analysis identified top entries such as ‘Endocytosis’, ‘Focal adhesion’, ‘Pathways in cancer’, and ‘Proteoglycans in cancer’ in common, which are well known to link to the biogenesis of EVs and the functions of EV protein cargo (Sung et al., 2021, Van Niel et al., 2018). The high efficiency and accuracy of EV protein identification were also evident from the high alignment rate of identified proteins with reported EV protein entries from Vesiclepedia (Figure 2c and Figure S1B).

In a comparative analysis, we found that integrins α2, α3, α5, αv, a6, β1, β4, and β5 were more abundant in CA1a EVs than in MB‐231 EVs (Figure 2d‐e) and that integrins α2, α3, αv, β1, β5, and β6 were enriched in 4T1 EVs as compared to 4TO7 EVs (Figure S1C‐D). Among these candidates, integrins α2, α3, αv, and β1 were commonly upregulated in metastatic EVs in both human and mouse pairs. Our findings corroborate a previous large‐scale proteomic study (Hurwitz & Meckes, 2019), where integrin profiles of EVs from sixty diverse human cancer cell lines, including breast cancer cells, were compared, revealing that levels of integrins αv, α6, and β1 correlate with cancer stage (Hurwitz & Meckes, 2019). Moreover, integrins αv and β1 are the most abundant alpha and beta integrin subunits in breast cancer‐derived EVs, respectively (Hurwitz & Meckes, 2019). Given these results, we focused on integrins αv and β1 (ITGAV and ITGB1) for further analysis.

### 2.3 Integrins αv and β1 are enriched in EVs from highly metastatic breast cancer cells

**FIGURE 3:**
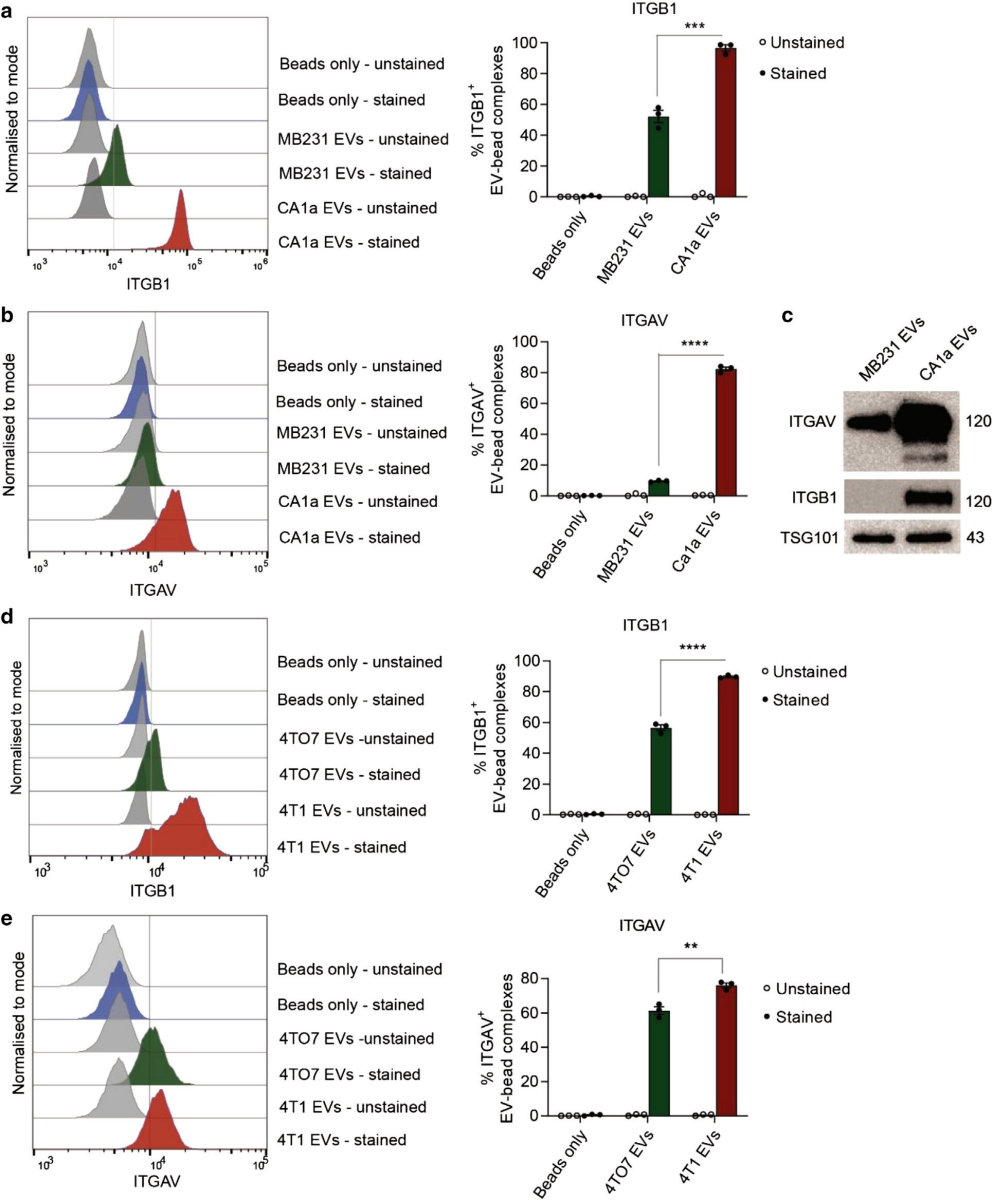
Integrin αv and β1 are more abundant on the surface of EVs from breast cancer cells of highly metastatic origin than EVs from cells of moderately metastatic potential. (a‐b) Flow cytometry analysis of integrin β1 and αv (ITGB1 and ITGAV, respectively) on the surface of EVs from human highly‐metastatic CA1a and poorly‐metastatic MB‐231 breast cancer cells, captured on anti‐human CD63 magnetic beads. Graphs present the average percentage of EV‐bead complexes with positive staining for ITGB1 and ITGAV in CA1a and MB‐231 EVs ± SEM. (c) Western blot analysis of ITGAV and ITGB1 in CA1a and MB‐231 EVs. TSG101 was used as a loading control. (d‐e) Flow cytometry analysis of ITGB1 and ITGAV on the surface of EVs from mouse highly‐metastatic 4T1 and poorly‐metastatic 4TO7 breast cancer cells, captured on anti‐mouse CD63 magnetic beads. Graphs present the average percentage of EV‐bead complexes with positive staining for ITGB1 and ITGAV in 4T1 and 4TO7 EVs ± SEM. Student t‐test **P ≤ 0.01, *** *P* ≤ 0.0001

To validate the levels of integrins αv and β1 on the surface of cancer‐derived EVs, we developed a flow cytometry‐based assay that allows us to detect surface proteins on EVs using affinity‐capture magnetic beads. In brief, magnetic beads pre‐conjugated with an antibody specific for human CD63, an established EV surface marker (Kalluri & Lebleu, 2020), were incubated with either CA1a EVs or MB‐231 EVs of equal particle counts. EV‐bead complexes were then stained with fluorescent dye‐conjugated antibodies and analysed using a flow cytometer. As we found that the anti‐human CD63 antibody on pre‐conjugated beads did not bind to mouse CD63, we performed an in‐house conjugation based on streptavidin‐biotin interaction (Sedlak et al., 2020), to produce mouse CD63‐capturing magnetic beads by incubating streptavidin‐conjugated magnetic beads with biotin‐labelled anti‐mouse CD63 antibody. We observed a higher level of ITGB1 (Figure 3a) and ITGAV (Figure 3b) in CA1a EVs than in MB‐231 EVs. The enrichment in ITGAV and ITGB1 in CA1a compared to MB‐231 EVs was confirmed by Western blot analysis (Figure 3c). The mouse EV pair from isogenic cells manifested a similar trend, that is, an upregulation of ITGB1 (Figure 3d) and ITGAV (Figure 3e) in 4T1 EVs in comparison to 4TO7 EVs. We excluded the possibility that the observations were induced by binding of fluorescent dye‐conjugated antibodies to magnetic beads or autofluorescence coming from EVs themselves, as groups of stained beads alone or unstained EV‐beads did not show significant background fluorescence (Figure 3a, b, d e). CD63 (Figure S2A), ITGB1 (Figure S2C), and ITGAV (Figure S2D) were abundant on the cell surface of breast cancer cells while CD11b was lacking (Figure S2B). We detected high percentages of CD63‐positive EV‐bead complexes, as a positive control, in all EV‐staining groups; while CD11b, a marker of myeloid cells that is not present in the cancer cell lines we used, was not detected (Figure S3A, B). The number of bead‐bound EV particles (equal to the number of input EVs less the number of unbound EVs in the supernatant) was approximately 2.3×10^8^ EV particles per 10 μg beads, with no significant difference between CA1a and MB‐231 EVs (Figure S4A‐B). We also compared ITGAV, ITGB1 and CD63 using a non‐reducing gel (to better detect CD63) followed by Western blotting (Figure S4C). CD63 levels were slightly higher in MB‐231 EVs albeit the molecular weight of CD63 was slightly different between the two cell lines which could be due to different patterns of glycosylation. The levels of ITGAV and ITGB1 proteins were clearly higher in CA1a EVs, consistent with our flow cytometry data and our Western blot data in Figure 3c (using reducing gel). Taken together, these data validated our proteomic analyses, and establish that integrins αv and β1 are upregulated in EVs from highly metastatic cells when compared to EVs from poorly metastatic cells.

### 2.4 Association of integrin αv with disease progression in breast cancer patients

**FIGURE 4:**
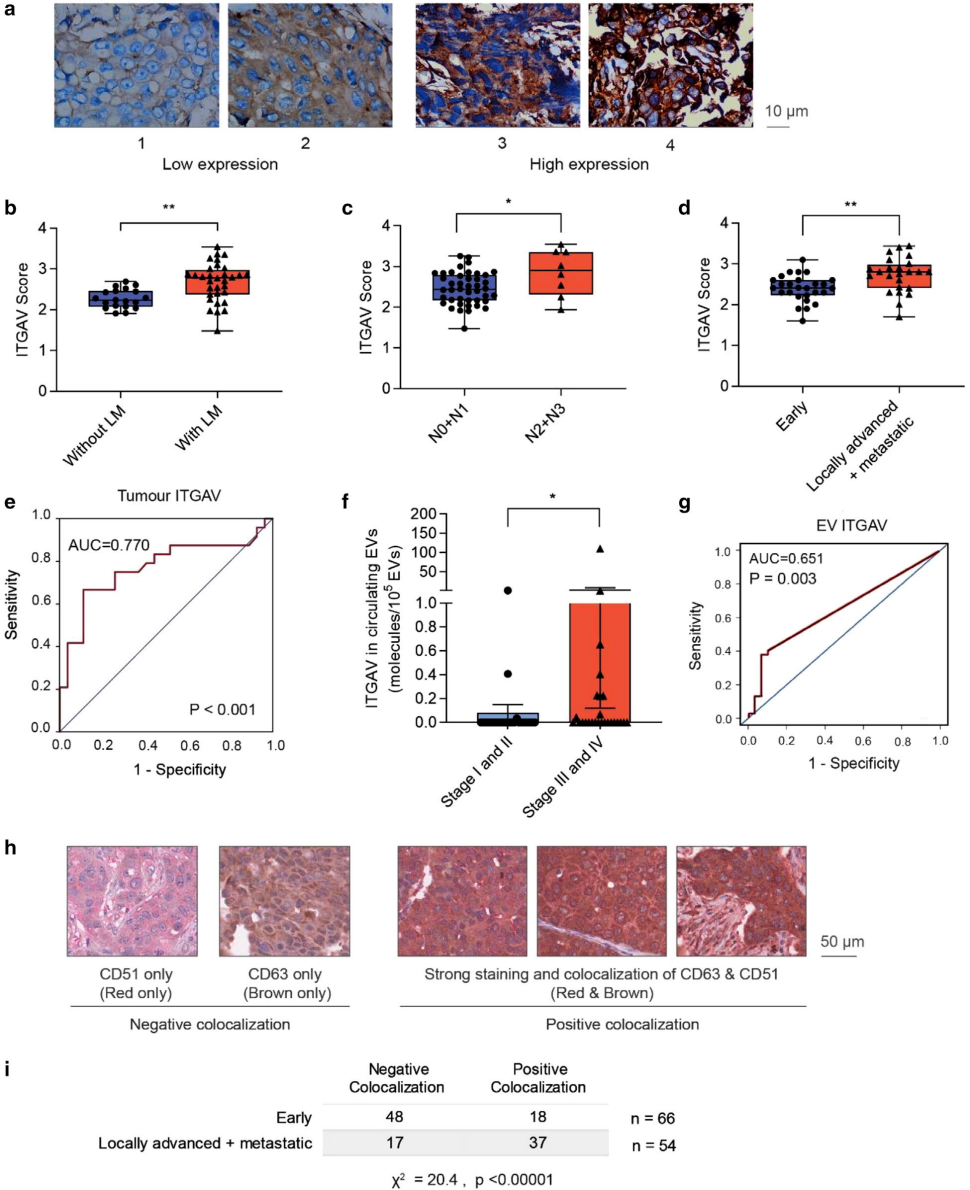
Integrin αv is associated with cancer progression in patients with breast cancer. (a) Scoring scheme and representative images from ITGAV IHC staining of tissue sections from patients with breast cancer. (b) Association of ITGAV expression score with the presence of lymph node metastasis (LM). (c) Association of ITGAV expression score with cancer N stages. (d) Association of ITGAV expression score with locally advanced and metastatic group (stage IIB ‐ IV) and early staged group (I‐IIA). (e) Receiver operating characteristic curve analysis of the diagnosis for locally advanced and metastatic group based on ITGAV expression score. (f) ELISA analysis of ITGAV protein in circulating EVs (number of ITGAV molecules per 10^5^ EVs) purified from blood samples of patients with early stage (I and II) and late stage (III and IV) breast cancer. (g) Receiver operating characteristic curve analysis of the diagnosis for early and late‐stage group based on ITGAV level in circulating EVs. Mann‐Whitney test *P ≤ 0.05, **P ≤ 0.01, ***P ≤ 0.001. (h) Representative IHC images of tumour sections stained for ITGAV (red) and CD63 (brown) from breast cancer patients. (I) ITGAV‐CD63 colocalization analysis using Chi‐square test

To better understand the clinical prognostic values of integrin αv, we examined the association of integrin αv with a set of clinicopathological parameters in patients with breast cancer using immunohistochemistry (IHC) of tumour sections. As we developed these assays, we were able to identify a suitable antibody for staining of ITGAV but not for ITGB1 despite testing an array of candidate antibodies. A total of 51 patients with breast cancer were eligible for study inclusion at the Guangxi Medical University hospital. ITGAV staining intensity was scored on a scale from 1 to 4 (low to high, respectively) (Figure 4a). The cohort reported an average score of 2.52 ± 0.44 (mean ± SD, same format for subsequent values in this section). We observed that patients with lymph node metastasis showed a significantly higher ITGAV score than those without (2.67 ± 0.47 vs. 2.27 ± 0.23; *P* = 0.001; Figure 4b). Moreover, analysis of node (N) stage, which evaluates the extent of lymph node metastasis, demonstrated that patients with more advanced N stage (N2 and N3) had a significantly increased ITGAV score as compared to those with N0 and N1 status (2.85 ± 0.57 vs. 2.46 ± 0.39; *P* = 0.022; Figure 4c). There were only two cases of patients with distant metastasis/M1 stage among the cohort of 51 patients, which is consistent with public statistics (Lord et al., 2012, Tosello et al., 2018). We pooled patients with locally advanced (TNM stage IIB to stage III) and metastatic (TNM stage IV) breast cancer and compared them with patients with early‐stage breast cancer (TNM stage I to IIA), with reference to the MD Anderson definition (Kantarjian et al., 2011). Patients with locally advanced and metastatic disease reported a statistically significant higher ITGAV score as compared to patients with early‐stage disease (2.75 ± 0.45 vs. 2.33 ± 0.34; *P* ≤ 0.001; Figure 4d). In addition, receiver operating characteristic (ROC) curve analysis illustrated that high ITGAV scores discriminate patients with locally advanced and metastatic breast cancer from patients with early‐stage breast cancer (area under curve = 0.770; 95% confidence interval = 0.631 ‐ 0.909; *P* = 0.001; Figure 4e). Other parameters examined, including age, T stage, tumour location (left or right), Ki‐67 index, p53 status, p16 status, and cytokeratin status, did not show a significant difference among groups. Collectively, these data demonstrated that high levels of integrin αv is indeed associated with disease progression in breast cancer patients.

In an independent study, we obtained blood samples from 56 breast cancer patients at the Singapore National University Hospital. Blood cells and debris were removed by centrifugation, and EVs were isolated from the supernatant using two rounds of ultracentrifugation. ITGAV was measured in the isolated EVs using ELISA. We were able to detect ITGAV in only three out of 30 patients (10%) with early‐stage cancer (stage I and II) but in nine out of 26 (34.6%) patients with late‐stage cancer (stage IIIA, IIIB and IV). The average level of ITGAV was significantly higher in circulating EVs of late stage cancer, compared to early‐stage cancer (Figure 4f). The ROC curve indicated that ITGAV in circulating EVs can be used as an indicator of late‐stage breast cancer although it is not as robust as the ITGAV IHC analysis (Figure 4g). The low rate of ITGAV detection in circulating EVs was due to the limited blood volume (0.5–1 ml per sample) available to us and the abundant blood‐cell‐derived EVs masked the detection of tumour‐derived EVs.

To confirm if ITGAV could be released in tumour‐derived EVs, we analysed the colocalization of ITGAV and CD63 in tumours using a tissue array collected from 128 breast cancer patients at the Singapore National University Hospital (Table 5). The analysis conducted by two independent certified pathologists indicated a high degree of ITGAV‐CD63 colocalization in 37 out of 54 patients (68.5%) with locally advanced and metastatic breast cancer but only 18 out of 66 patients (27.3%) with early‐stage breast cancer indicating a significant difference between the two groups (χ^2^ = 20.4, *P* < 0.00001) (Figure 4h‐i). Of note, ITGAV was very low in normal adjacent breast tissues collected from four anonymized breast cancer patients although CD63 was high in all of these samples (Figure S4D). CD63 is a marker of multivesicular bodies, the major endosomal compartment giving rise to CD63^+^ EVs. Thus, the higher colocalization of CD63 and ITGAV suggests a higher probability of ITGAV export into EVs in advanced breast cancer. The significant association between ITGAV‐CD63 colocalization with locally advanced and metastatic breast cancer also suggests a prognostic value for this co‐staining, which can be used to predict metastatic probability.

### 2.5 Integrin αv is elevated in circulating EVs from xenografted mice bearing metastatic tumours

**FIGURE 5:**
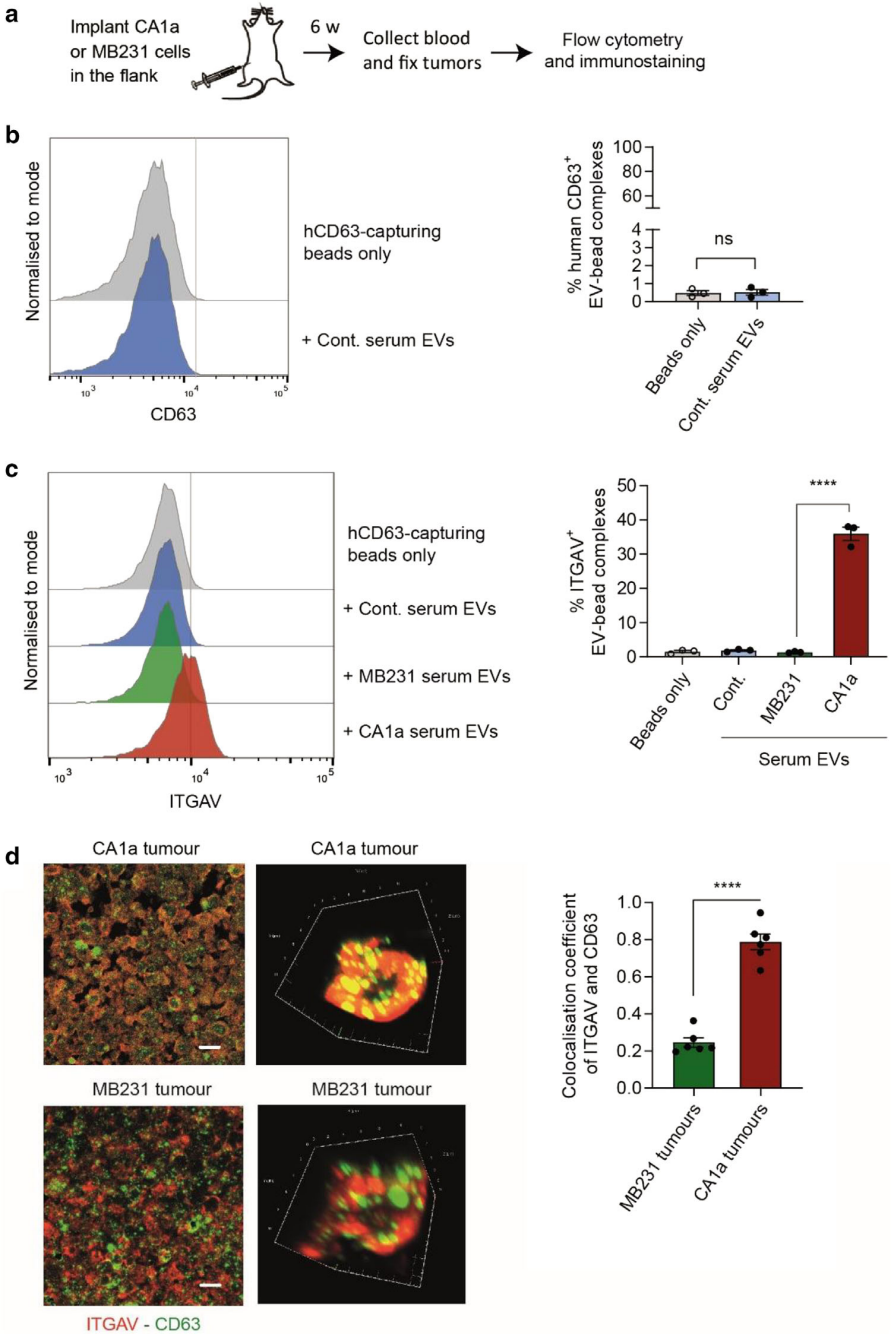
Integrin αv is enriched in circulating EVs from xenografted mice bearing metastatic breast cancer tumours. (a) Schema of implanting CA1a or MB‐231 cells in the flank of NSG mice for circulating EV analysis and confocal imaging. (b) Flow cytometry analysis of anti‐human CD63 beads incubated with EVs from untreated NSG mouse serum (control, cont.). (c) Flow cytometry analysis of ITGAV on EVs from the serum of NSG mice bearing CA1a tumour, MB‐231 tumour or no tumour in the flank (cont), captured by anti‐human CD63 beads. Serum from two mice in the same group were pooled (*n* = 3 paired groups of mice). (d) Representative images and colocalization analysis of ITGAV and CD63 in CA1a and MB‐231 tumours. Xenografted tumour sections were stained for CD63 and ITGAV. Images and 3D projection were obtained using confocal microscopy to identify colocalized staining and calculate the colocalization coefficient index (*n* = 6 mice). Student's t‐test, ****P ≤ 0.0001.

To investigate whether metastatic cells secrete EVs bearing increased ITGAV into the circulation in vivo, we implanted an equal count of either CA1a cells or MB‐231 cells into the flanks of NSG mice. Blood was collected for isolation of circulating EVs, and tumours were fixed and sectioned for immunofluorescence staining. Mice were sacrificed when the tumour reached 15 mm at the largest dimension (Figure 5a). Circulating EVs of two mice from the same group were pooled to improve EV recovery. We confirmed that anti‐human CD63 magnetic beads did not capture mouse circulating EVs from uninjected NSG mice (Figure 5b). Next, we incubated anti‐human CD63 magnetic beads with circulating EVs from un‐injected, MB‐231‐implanted, and CA1a‐implanted mice, and found that only circulating EVs from CA1a‐implanted mice exhibited increased levels of ITGAV (Figure 5c). To confirm this result, we co‐stained the tumour sections with fluorescent anti‐human CD63 and anti‐human ITGAV antibodies and examined them using confocal microscopy. Colocalization coefficients were then calculated with a coefficient closer to one indicating more colocalization. We observed that CA1a tumours had more localisation of CD63 and ITGAV than MB‐231 tumours (Figure 5d), supporting our finding that increased ITGAV was exported into the cancer‐derived EVs in metastatic tumours in vivo.

### 2.6 Galectin 3 mediates the export of integrin αv into EVs from metastatic cells

**FIGURE 6:**
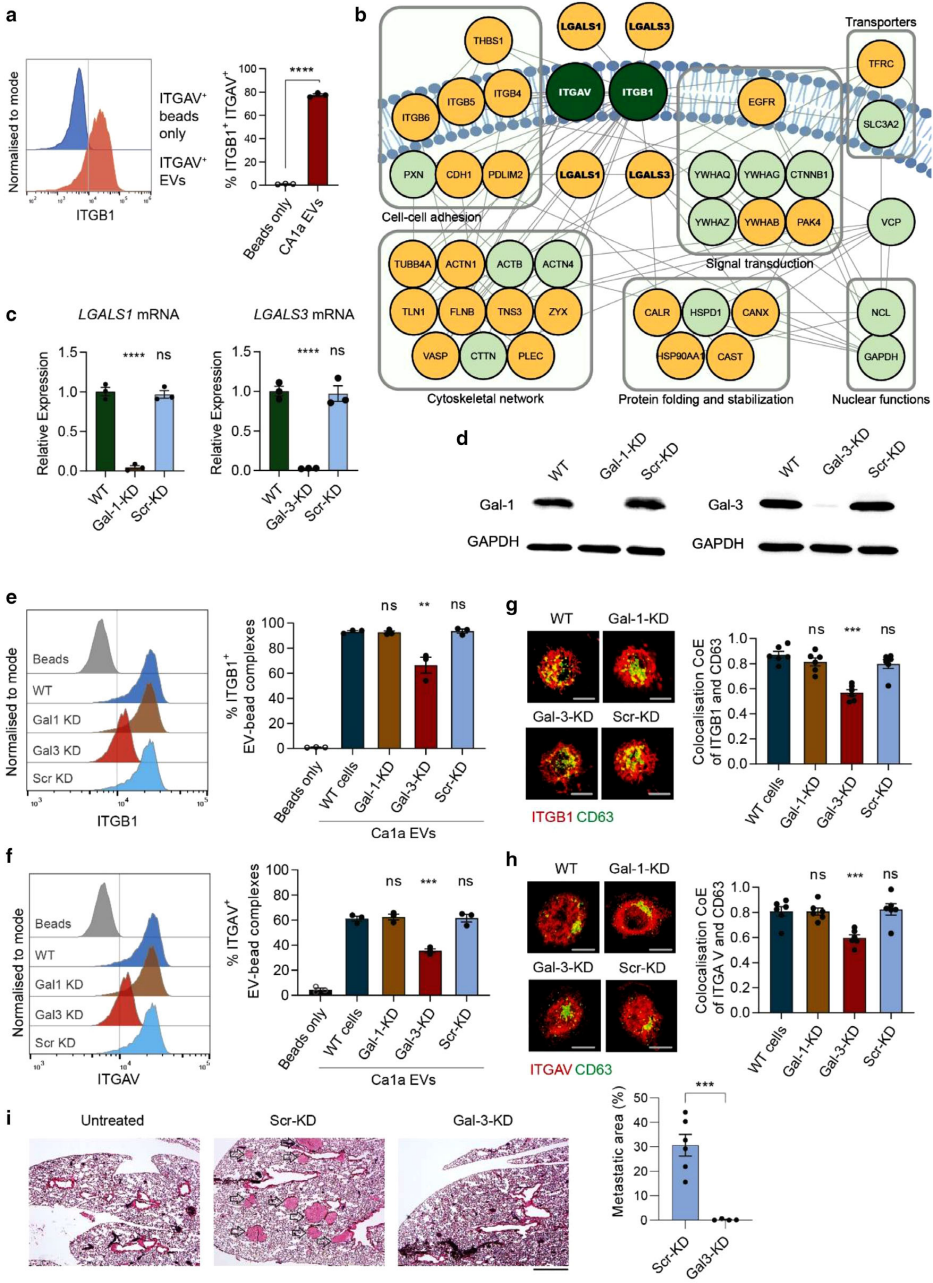
Galectin‐3 mediates the export of integrin αv and β1 into CA1a EVs. (a) Flow cytometry analysis of ITGB1 on ITGAV^+^ EVs from CA1a cells, captured by anti‐human ITGAV beads. (b) Proteins associated with ITGAV in CA1a cells and MB‐231 cells, identified using immunoprecipitation and mass spectrometry with a biotinylated ITGAV antibody and immobilized streptavidin magnetic beads. Shown in the map are known protein‐protein interactions among the most abundant ITGB1‐ and ITGAV‐associated proteins Yellow nodes indicate proteins with higher enrichment in the ITGAV immunoprecipitation of CA1a cells compared to MB‐231 cells. (c‐d) qRT‐PCR and Western blot analysis of galectin‐1 and ‐3 (Gal‐1/3 encoded by *LGALS1/3*) in CA1a cells transduced with shRNAs targeting Gal‐1/3 or a scrambled (Scr) control shRNA. (e‐f) Flow cytometry analysis of ITGB1 and ITGAV on CA1a EVs after knockdown of Gal‐1 and Gal‐3, captured by anti‐human CD63 beads. (g‐h) Representative confocal microscopy images and colocalization coefficient (CoE) analysis of ITGB1 and ITGAV with CD63 in CA1a cells after knockdown of Gal‐1 and Gal‐3 (*n* = 6). (i) H & E staining of lung sections from NSG‐SGM3 mice injected (i.v.) with CA1a cells transduced with the scrambled or Gal‐3 shRNAs, compared to untreated mice. Metastatic nodules are indicated by the arrows. Scale bar: 500 μm. The graph presents the percentage of lung area with metastasis (*n* = 4–6). Student's t‐test ** *P* ≤ 0.01, *** *P* ≤ 0.001, ns: non‐significant

To confirm the association of ITGAV with ITGB1 in EVs derived from CA1a cells, we produced in‐house conjugated anti‐human ITGAV magnetic beads and precipitated ITGAV^+^ CA1a EVs. We observed a high abundance of ITGB1 in the ITGAV^+^ EVs, suggesting a strong association of ITGAV and ITGB1 in CA1a EVs (Figure 6a). ITGB4 and ITGB5 were also detected albeit at lower levels on ITGAV^+^ EVs from CA1a cells (Figure S5A). Next, to identify the proteins that potentially regulate the export of ITGAV into CA1a EVs and MB‐231 EVs, we performed immunoprecipitation (IP) to pull down ITGAV and associated proteins in CA1a cell lysates and used mass spectrometry for protein identification. GO analysis of the IP and co‐IP proteins from ITGAV pull‐down in CA1a EVs showed ‘cell‐cell adhesion’ and ‘cadherin binding involved in cell‐cell adhesion’ among the top‐ranked entries in biological processes and molecular functions, reflecting the broadly known function of ITGAV in cell adhesion (Table S3). Moreover, in the GO analysis of cellular components, top entries included ‘extracellular exosome’, ‘membrane’, and ‘extracellular space’, implying the potential abundant export of ITGAV and associated proteins into EVs (Table S4). When mapping the ITGAV‐IP and co‐IP proteins onto protein‐protein interaction (PPI) databases, we established a PPI network consisting of 39 proteins (Figure 6b). Our attention was drawn to two candidate proteins, galectin‐1 (Gal‐1) and galectin‐3 (Gal‐3), in the network, which are known to be glycosylation‐dependent interactors of ITGB1 (Obermann et al., 2017), the integrin beta subunit associated with ITGAV in CA1a EVs. Gal‐1 and Gal‐3 belong to the β‐galactoside‐binding family of galectins and are reported to regulate integrin shuttling in cells (Fortin et al., 2010, Hönig et al., 2018). Importantly, Gal‐1 and Gal‐3 were lacking in parallel ITGAV pull‐downs using in MB‐231 cells, the parental cells of low metastatic potential that give rise to MB‐231 EVs with low abundance of ITGB1 and ITGAV.

To investigate whether Gal‐1 and Gal‐3 regulate the export of ITGAV to CA1a EVs, we knocked down *LGALS1* and *LGALS3*, which encode Gal‐1 and Gal‐3, respectively, in CA1a cells using short hairpin RNAs (shRNA). Four shRNA sequences were pre‐selected for *LGALS1* and *LGALS3*, respectively, from the Broad Institute RNAi Consortium (Root et al., 2006), based on predicted efficacies, and cloned into a lentiviral vector. Our pre‐selected shRNA sequences demonstrated high efficiency in knocking down *LGALS1* and *LGALS3* (Figure S5B‐C). We selected the most efficient shRNA target sequences to knock down *LGALS1* and *LGALS3*. qPCR and western blot analysis showed that the shRNA‐mediated knockdown led to a significant reduction of *LGALS1* and *LGALS3*, at both mRNA and protein levels, as compared to wild‐type or negative control using scrambled shRNA (Figure 6c‐d). Knockdown of either *Gal‐1* or *Gal‐3* did not cause a significant change in mRNA and protein levels of *ITGB1* or *ITGAV* in CA1a cells, as shown by qPCR and Western blotting (Figure S5D‐G). However, *Gal‐3* knockdown led to a minor reduction of ITGB1 and ITGAV on the surface of CA1a cells, as shown by flow cytometry analysis (Figure S5H‐I). We then used our affinity‐based magnetic beads to capture the CD63^+^ subset of EVs from wild‐type and knockdown CA1a cells and analysed the EV surface for ITGB1 and ITGAV. Flow cytometry data showed that knockdown of Gal‐3 but not Gal‐1 led to a reduction of EV surface levels of ITGB1 and ITGAV in the CD63^+^ subset of CA1a EVs (Figure 6e‐f), further confirmed by decreased colocalization of CD63 and ITGB1 or ITGAV in Gal‐3 knockdown cells (Figure 6g‐h). To test if Gal‐3 is important for breast cancer metastasis, we injected CA1a cells treated with scrambled and Gal‐3 shRNA into the tail vein of immunodeficient NSG‐SGM3 mice. After 9 weeks, we observed many metastatic nodules in the lung of the mice bearing CA1a with scrambled shRNA but very few nodules were found in the mice injected with CA1a cells subjected to Gal‐3 knockdown (Figure 6i). These data demonstrate that Gal‐3, in part, contributes to the export of integrin αvβ1 into CA1a EVs and thereby plays an important role in breast cancer metastasis.

### 2.7 Integrin αvβ1 mediates EV binding to fibronectin in the extracellular matrix

**FIGURE 7:**
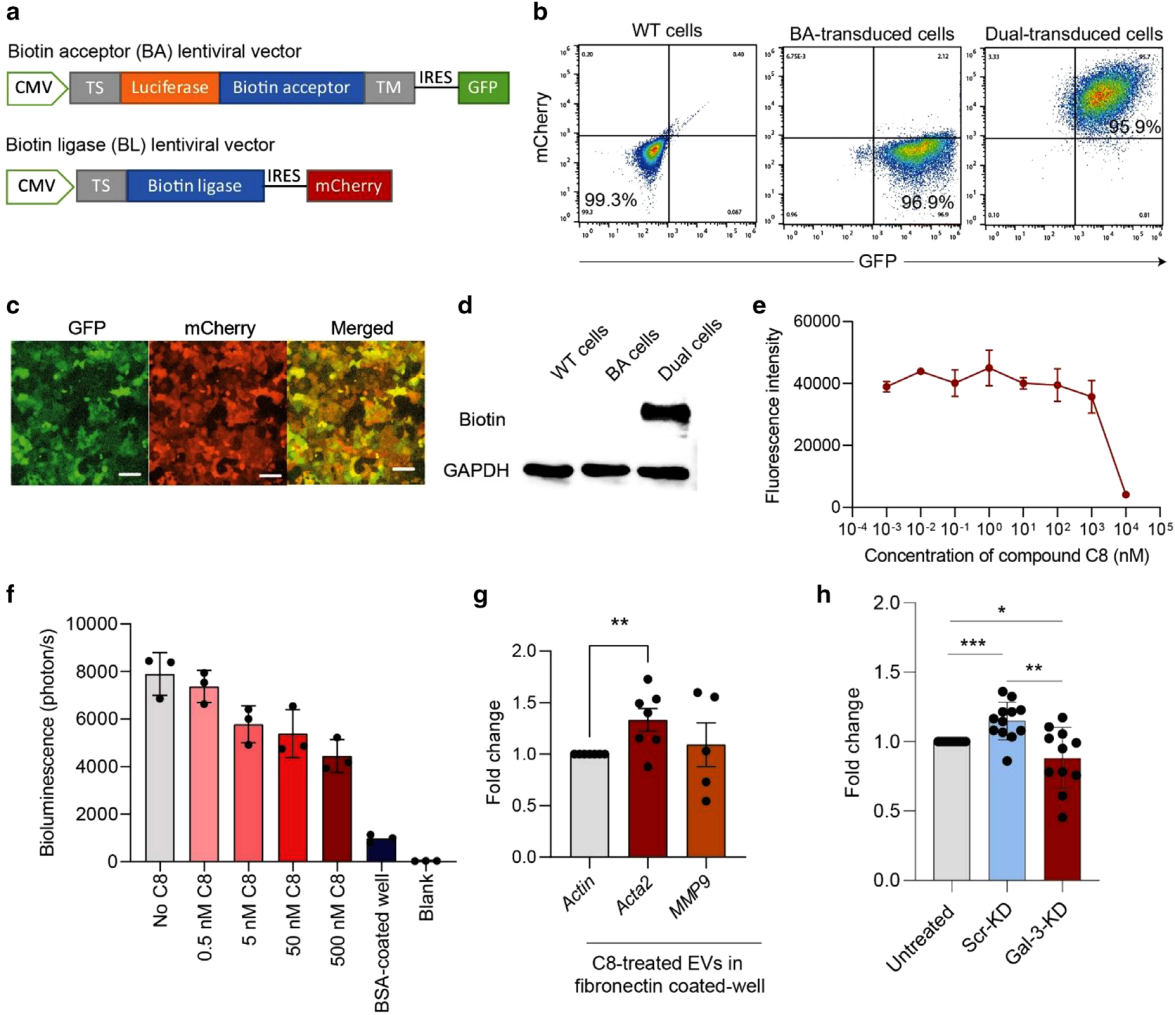
ITGAV‐B1 complex is essential for fibronectin‐dependant EV retention. (a) Schema of lentiviral vectors transduced in CA1a cells to allow for gaussia luciferase and biotin expression on EV surface. TS, targeting signal. TM, transmembrane domain. IRES, internal ribosomal entry site. (b‐c) Dual‐transduction led to dual GFP and mCherry florescence in CA1a cells as shown by flow cytometry and fluorescent microscopy, respectively. (d) Western blot analysis showing the expression of biotin in CA1a cells after dual transduction. (e) Fluorescent signals of CFSE‐labelled CA1a cells that adhered on a fibronectin‐coated plate after a treatment with increasing concentration of C8 compound which blocks the integrin αvβ1 complex. (f) Bioluminescent signal of CA1a‐BA EVs (10^10^ particles/ml) adhered to a fibronectin‐coated plate under the influence of C8 in a dose‐dependent manner. Adhesion of CA1a‐BA EVs to a BSA‐coated plate is included as a negative control. (g) qPCR analysis of CAF markers, *Acta2* and *MMP9* in mouse primary fibroblasts after 72‐h incubation in fibronectin‐coated plate pretreated with CA1a EVs (the plate was incubated with 5×10^9^ EVs and washed twice). (h) qPCR analysis of *Acta2* in mouse primary fibroblasts after 72‐h incubation in fibronectin‐coated plate pretreated with EVs from CA1a cells transduced with scrambled or Gal‐3 shRNA (KD). *Acta2* and *MMP9* expression in EV treated cells were normalized to beta‐actin and presented as fold change relative to the untreated control. Student's t‐test, *P ≤ 0.05, **P ≤ 0.01, *** *P* ≤ 0.001. Each data point represents a biological replicate

To facilitate the tracing of CA1a EVs, we engineered the surface of CA1a EVs with a labelling system based upon Gaussia luciferase and biotin dual reporters (Lai et al., 2014). This system features two lentiviral vectors, namely, a biotin acceptor (BA) vector and a biotin ligase (BL) vector (Figure 7a). The BA vector contains a membrane targeting signal that recruits the Gaussia luciferase biotin acceptor fusion protein to the EV membrane, while the BL vector catalyzes biotin addition to the biotin acceptor site. BA and BL vectors also express separate green fluorescent protein (GFP) and mCherry as transduction reporters. CA1a cells co‐transduced with BA and BL lentiviral vectors demonstrated strong signals of dual GFP and mCherry fluorescence, as determined by flow cytometry and fluorescent microscopy, respectively, indicating the high efficiency of dual transduction (Figure 7b‐c). Dual transduced cells express abundant biotin as shown by western blot detection using streptavidin (Figure 7d).

Given the role of integrin αvβ1 in fibronectin‐dependant cell adhesion (Zhang et al., 1993), we wondered whether integrin αvβ1 on the EV surface plays a role in EV binding to fibronectin in the extracellular matrix. To block integrin αvβ1, we used a potent and highly specific small‐molecule inhibitor, Compound 8 (C8) (Reed et al., 2015). Blocking integrin αvβ1 inhibited the adhesion of CA1a cells to fibronectin‐coated plates, as indicated by the reduction of fluorescence signal from CFSE cell labelling when adding C8 (Figure 7e). This result confirmed the presence of integrin αvβ1 on the surface of CA1a cells and established that one function of integrin αvβ1 is to mediate fibronectin‐dependent cell adhesion. We next blocked integrin αvβ1 on the surface of CA1a‐BA EVs with C8 and used bioluminescence from Gaussia luciferase surface labelling as a readout to quantify the retention of EVs in fibronectin‐coated plates. We found a dose‐dependent decrease of bioluminescence from Gaussia luciferase‐labelled EVs with increased C8 concentration (Figure 7f). Subsequently, we pretreated a fibronectin‐coated plate with CA1a EVs and washed the plate twice before adding mouse primary fibroblasts to the plate. After 72 h, we found a significant increase in *Acta2*, which encodes the CAF marker αSMA (Figure 7g). Moreover, knockdown of *Gal‐3* abolished the effect of CA1a EVs on *Acta2* upregulation in fibroblasts, suggesting that Gal‐3 mediated EV production plays a role in fibroblast differentiation (Figure 7h). The results are consistent with our previous study in which we demonstrated the role of breast cancer EVs in promoting fibroblast differentiation into CAFs and how CAFs contribute to cancer metastasis (Vu et al., 2019). Taken together, the above data demonstrated the important role of surface integrin αvβ1 in EV binding to fibronectin in the extracellular matrix.

## 3 DISCUSSION

Over the past decade, we have seen compelling evidence that establishes the multifaceted, instrumental functions of EVs in tuning the tumour microenvironment, especially in promoting metastasis, as we recently reviewed (Lima et al., 2021, Mo et al., 2020) and previously investigated (Le et al., 2014, Vu et al., 2019) in agreement with other studies (Armacki et al., 2020, Shinde et al., 2020, Zomer et al., 2015). A seminal work by Hoshino et al (Hoshino et al., 2015) accentuates the cardinal roles of EV‐bound integrins in mediating the preferential uptake of cancer‐derived EVs by stromal cells for pre‐metastatic niche establishment. Indeed, altered integrin expression has frequently been described in various cancers and the versatility and complexity of diverse integrins make these alternations influence every step of cancer progression and metastasis (Hoshino et al., 2015). Here, we identified a panel of upregulated EV‐bound integrins from comparative mass spectrometry‐based profiling of EVs from breast cancer cells of different metastatic potentials. Among these integrin candidates, integrin αv and β1 were of special interest to us as they were consistently upregulated in both human and mouse EVs of highly metastatic origin. Our proteomic data were supported by a large‐scale proteomic study where integrins αv and β1 were found as the most abundant alpha and beta integrin subunits, respectively, in breast cancer‐derived EVs (Hurwitz & Meckes, 2019). Moreover, increased EV‐bound integrin αv and β1 are pan‐cancer markers that correlate with advanced cancer stages (Hurwitz & Meckes, 2019). Our results also showed the consistent upregulation of integrin αv and β1 in both human and mouse EVs of highly metastatic origin.

We validated the upregulation of integrin αv and β1 in both human and mouse metastatic EVs using an affinity‐capturing, magnetic bead‐based flow cytometry protocol. As EVs frequently contain a large proportion of particles below 200 nm in diameter, direct analysis of single EVs using conventional flow cytometers is often challenging as the particles are smaller than the wavelength of lasers and EVs, with much fewer surface molecules compared to cells, and thus generate less signal (Hamidi & Ivaska, 2018, Lannigan & Erdbruegger, 2017). Although there are now advanced imaging flow cytometers that can analyse smaller particles, complex optimization is required, and the availability of advanced equipment also limits the current utility of such approaches (Hamidi & Ivaska, 2018, Lannigan & Erdbruegger, 2017). Our magnetic bead‐based method combines the advantages of both, capturing EVs based on affinity specific to EV surface markers and amplifying the physical size and the fluorescence signal of EVs to be analysed using conventional flow cytometers.

Our approach to bead‐aided EV flow cytometry could also detect integrin αv on circulating EVs in xenografted mice. Importantly, the higher level of integrin αv on EVs was able to predict the metastatic potential, which relied on several attributes. We used anti‐human CD63 magnetic beads to capture cancer‐derived EVs of human origin in mouse circulating EVs, since the anti‐human CD63 magnetic beads did not recognize mouse CD63. Furthermore, CD63 is enriched in EVs from endosomal pathways (Görgens et al., 2019). The mice we selected were NSG, which are commonly used in xenografted models and lack a substantial set of T, B, and NK cells (Sung et al., 2020). These immune cells are all reported to produce CD63^+^ EVs (Okoye Isobel et al., 2014, Oksvold et al., 2014, Shultz et al., 2019), while EVs from mature red blood cells are mostly plasma membrane‐derived (Zhang et al., 2019). Depleting immune cells contributed to reducing background mouse EVs. This design enabled us to detect human integrin αv on cancer‐derived EVs from mouse circulating EVs. However, this approach, based on species‐specific reactivity, would not be applicable to detecting cancer‐derived EVs in circulation in humans as many other cell types also secrete human CD63^+^ EVs into the circulation and such EVs would thereby confound the results. Hence, finding EV surface markers specific to cancer‐derived EVs will facilitate the future translation of this method.

Despite not being able to capture and detect cancer‐derived EVs from circulating EVs in patient samples, we purified the total EV population from patients’ blood and compared the level of ITGAV using ELISA. Interestingly, integrin αv was detected more frequently in circulating EVs of patients with late‐stage (III and IV) than early‐stage (I and II) breast cancer. We cannot be certain whether the detectable integrin αv originated exclusively from tumour cells in the patients. However, our data suggest that integrin αv levels in total‐blood EVs can be used as a prognostic marker. Furthermore, we demonstrated that integrin αv is associated with breast cancer progression from IHC analysis of tumour sections. Indeed, the association of elevated integrin αv levels with progression and poor prognosis in patients has been reported in many solid tumours, including colorectal cancer (Di Pace et al., 2020), gastric cancer (Waisberg et al., 2014), and esophageal adenocarcinoma (Wang et al., 2019). Targeting integrin αv reduced metastasis in a breast cancer mouse model (Loeser et al., 2020). In particular, we observed integrin αv overexpression in breast cancer patients with lymph node metastasis and advanced N stage, consistent with an earlier report on integrin αv‐related lymph node metastasis in patients with laryngeal and hypopharyngeal carcinoma (Cheuk et al., 2020). In addition, we also found that colocalization of integrin αv and CD63 was highly associated with lymph‐node and distant metastasis in breast cancer patients, consistent with ITGAV‐CD63 colocalization in xenografted CA1a tumours. Together with our in vitro and in vivo data, this observation highlights EV‐bound integrin αv as a potential novel target for prognostics and therapeutics in breast cancer.

We observed that knockdown of Gal‐3 in CA1a cells led to the downregulation of integrin αv and β1 in CD63^+^ EVs, a result supported by decreased colocalization of CD63 with integrin αv and β1 following knockdown. Since integrin β1 is an abundant binding partner in integrin‐αv‐positive CA1a EVs, we hypothesized that the export of integrin αv to EVs is dependent on integrin β1. Our findings were consistent with previous studies that illustrated interactions between Gal‐3 and integrin β1 (Hönig et al., 2018, Lu et al., 2011). Furtak et al demonstrated that Gal‐3 mediates endocytosis of integrin β1 in breast cancer cells (Lu et al., 2011) and Hönig et al. reported that knockdown of Gal‐3 reduced integrin β1 tethering to the apical plasma membrane in epithelial cells (Hönig et al., 2018). As reviewed by us and others (Kalluri & Lebleu, 2020, Zhang et al, 2021), there are many steps that can allow protein cargo to be exported into exosomes. Export can derive from delivery and tethering of protein cargo to the membrane followed by endocytosis of the cargo, an initial step in exosome biogenesis. Later processes, such as interactions of early endosomes with other membrane‐bound organelles and formation of intraluminal vesicles through inward invagination of late endosomal membrane, provide opportunities for additional cargo to be exported into EVs (Kalluri & Lebleu, 2020, Zhang et al, 2021). As Gal‐3 mediates two out of these important steps, delivery and tethering of integrin β1 to membrane and endocytosis of integrin β1, it makes sense that knockdown of Gal‐3 would cause a reduction of integrin β1 together with the partnered integrin αv in CD63^+^ EVs. Furthermore, Gal‐3 is found to recruit the ESCRT component ALIX, another common EV biogenesis player and EV marker, to repair Leu‐Leu‐OMe‐induced lysosomal membrane damage (Furtak et al., 2001). In a PPI network of overexpressed proteins in CA1a EVs compared to MB‐231 EVs, we noticed the presence of galectin‐3‐binding protein (LGALS3BP), which is known to interact with both ITGAV/B1 (Jia et al., 2020) and Gal‐3 (Rosenberg et al., 1991, Stampolidis et al., 2013), a result that could imply an interaction network among these three proteins. We achieved high Gal‐3 knockdown efficiency, but this led only to a partial reduction of integrin αvβ1 in CD63^+^ EVs. This result likely indicates that Gal‐3 only contributes to the control of ITGAV/B1 export to EVs in part and that other mechanisms or alternative pathways likely also facilitate the export.

One of the known ligands of integrin αvβ1 is fibronectin (Koths et al., 1993), which is a key glycoprotein in the cancer‐related extracellular matrix (Zhang et al., 1993) and can bind to integrins via the RGD motif recognition to mediate cell adhesion (Saad et al., 2002). Given this, we applied an integrin‐αvβ1‐specific small‐molecule inhibitor to block integrin αvβ1 on EVs (Reed et al., 2015) and observed a dose‐dependent reduction of EV attachment to fibronectin. This experiment mimicked the cancer‐associated extracellular microenvironment that involves fibronectin and integrin αvβ1 and suggested that ITGAV/B1 may be crucial to the fibronectin‐dependent retention of metastatic EVs in the tumour microenvironment. We further showed that EVs retained on fibronectin were taken up by fibroblasts, which differentiated into CAFs. We and others have also shown that CAFs are important for tumour growth and metastasis. Hence, the role of integrin αvβ1 in mediating EV retention has an indirect effect on CAF differentiation and metastasis. Strikingly, we observed that knockdown of Gal‐3 led to a significant decrease in metastatic colonization of CA1a cells, supporting the role of Gal‐3 in mediating integrin αvβ1 export and mechanisms related to metastasis.

**FIGURE 8:**
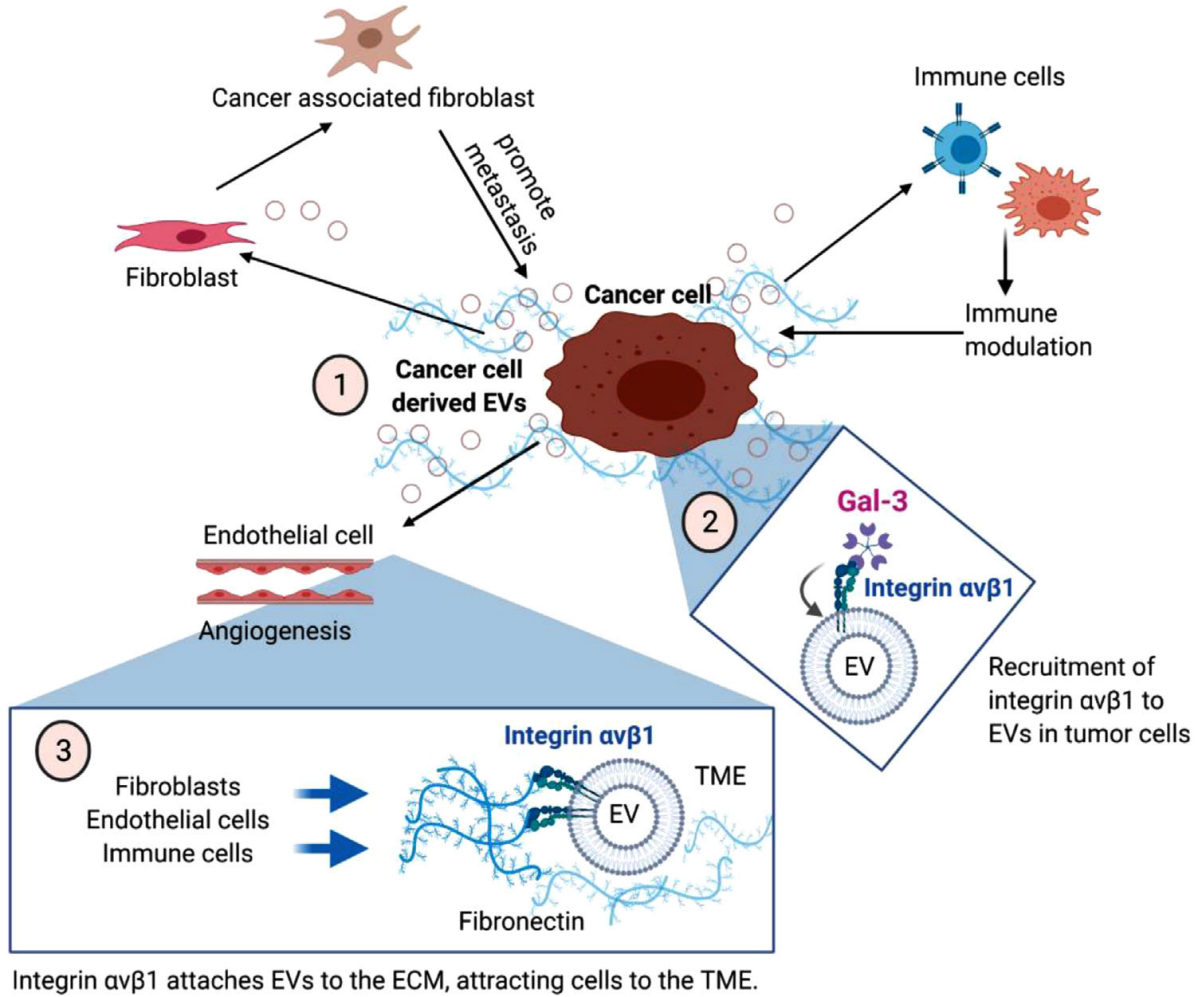
A working model for integrin αvβ1 in the tumour microenvironment. (1) Breast cancer cells secrete EVs to mediate their communication with other cell types, including fibroblasts, endothelial cells and immune cells, in the tumour microenvironment (TME). The TME cells in turn support tumour growth, angiogenesis and metastasis. (2) EVs from metastatic breast cancer cells are enriched in integrin αvβ1 complex that is sorted into EVs by Gal‐3 in the cells. (3) Integrin αvβ1 attaches EVs to fibronectin in the extracellular matrix (ECM) that may help to attract more host cells into the TME

In summary, our study demonstrates that integrin αvβ1 is linked to EVs from metastatic breast cancer cells, whose export into EVs is in part regulated by Gal‐3 (Figure 8). Additionally, ITGAV/B1 is important to the fibronectin binding and retention of metastatic EVs, which we previously demonstrated to be taken up by a variety of stromal cells in the tumour microenvironment, including fibroblasts, endothelial cells, and immune cells (Vu et al., 2019) (Figure 8). It is increasingly accepted that the tumour is a complex conglomerate of cancer cells, stromal cells, EVs, and extracellular matrix, which act in concert for the tumour to metastasise and progress (Lima et al., 2021). Thus, combination/comprehensive strategies targeting multiple aspects of tumour biology are now in progress and in use to treat cancer and these approaches are shifting the therapy paradigm (Johansson, 1997, Nguyen et al., 2021). We hope that our study contributes a valuable piece to solve the bigger puzzle of the tumour microenvironment through understanding the implications of EV‐bound integrin αvβ1 in breast cancer metastasis.

## 4 MATERIALS AND METHODS

### 4.1 Cell culture

#### 4.1.1 Tissue culture conditions and cell line origins

As previously described (Vu et al., 2019), cells were cultured in DMEM (Thermo Fisher, USA) supplemented with 10% foetal bovine serum (FBS) (Biosera, France), 1× penicillin‐streptomycin (pen‐strep) (Thermo Fisher, USA), and 1× Plasmocin prophylactic (InvivoGen, USA) and incubated at 37°C with 5% CO_2_. Human embryonic kidney (HEK)‐293T cells and human breast cancer MDA‐MB‐231 (MB‐231) cells were purchased from ATCC. Human breast cancer MCF10CA1a (CA1a) cells and mouse breast cancer 4T1 and 4TO7 cells were obtained from Karmanos Cancer Institute (Wayne State University, USA).

#### 4.1.2 Establishment of luciferase cell lines for in vivo metastasis monitoring

CA1a and MB‐231 cells were transduced with a lentivirus vector expressing surface membrane‐bound firefly luciferase (pLV‐Fluc‐SmCherry‐Puro) modified with a CAG promoter, as previously described (Le et al., 2014, Vu et al., 2019). CA1a‐luc and MB‐231‐luc cells were selected with 3 μg/ml puromycin (Thermo Fisher, USA) for 2 weeks and sorted using fluorescence‐activated cell sorting (FACS) twice to establish stable cell lines.

#### 4.1.3 Establishment of galectin‐knockdown cell lines using shRNA

shRNA sequences were obtained from the Broad Institute RNAi Consortium (Root et al., 2006), selected based on algorithm‐predicted scores, and cloned into a proprietary lentiviral vector featured with dual selection markers of copGFP and puromycin resistance (Guangzhou IGE Biotech, China). A non‐targeting scrambled shRNA sequence was cloned into the same vector and used in parallel as a negative control. shRNA sequences were summarised in Table 4. Transduced CA1a cells were selected and maintained as described above.

#### 4.1.4 Establishment of CA1a‐BA‐BL cells

Biotin acceptor (BA) lentiviral vector was obtained from Prof. Xandra Breakefield (Harvard Medical School, USA) and Dr. Charles Lai (Academia Sinica, Taiwan), and biotin ligase (BL) lentiviral vector was purchased from Vector Core Facility of Massachusetts General Hospital, USA (Lai et al., 2014). CA1a cells were co‐transduced with BA and BL vectors, and later selected and maintained as described above.

### 4.2 EV production and assays

#### 4.2.1 EV purification using flotation ultracentrifugation and FPLC‐SEC

FBS was diluted in DMEM and ultracentrifuged at 120,000 ×g for 18 h at 4°C using a SW32Ti rotor (Beckman Coulter, USA) for EV depletion, 0.22‐μm‐filtered, and then further diluted with DMEM to create a 10% FBS‐supplemented medium with 1× pen‐strep for the purpose of EV production, as previously described (Vu et al., 2019). We cultured mouse or human breast cancer cells with an EV‐depleted medium and collected the conditioned medium (CM). CM went through three rounds of low‐speed differential centrifugation to remove dead cells and then was 0.45‐μm‐filtered. 25 ml of clarified CM was layered on top of a 2 ml 60% sucrose cushion (Acros Organics, USA) and ultracentrifuged at 100,000 ×g for 90 min at 4°C using a SW32Ti rotor (Beckman Coulter, USA). We collected the interface of ∼3 ml between the sucrose cushion and CM. We then layered 10 ml of the interface from the first‐round flotation ultracentrifugation on top of a 0.75 ml 60% sucrose cushion and ultracentrifuged them at 110,000 ×g for 12 h at 4°C using a SW41Ti rotor (Beckman Coulter, USA). The interface of ∼1 ml was collected in each tube from the second‐round flotation ultracentrifugation. A prepacked qEV10 SEC column (Izon Science, New Zealand) was connected to the NGC Quest 10 Chromatography System (BioRad, USA). We equilibrated and eluted the samples with 0.22‐μm‐filtered, degassed PBS in 75 fractions (2 ml/fraction) at 4°C. Fractions were collected individually for nanoparticle tracking analysis and BCA assay. Absorbance at 280 nm was obtained from the integrated ChromLab Software (BioRad, USA). EV‐enriched fractions 8–14 were combined, 0.45‐μm‐filtered and loaded onto an Amicon Ultra‐15 centrifugal filter unit (MilliporeSigma, USA) and centrifuged at 3260 ×g at 4°C for further concentration, as reported earlier (Vu et al., 2019).

#### 4.2.2 Isolation of EVs from patient whole blood samples

Whole blood samples from patients with breast cancer at various stages were collected by clinicians at the National University Hospital with informed consent. Sample processing was performed according to the approval of the Institutional Review Board at the National University of Singapore. Frozen samples were transferred into new 1.5 ml Eppendorf tubes and centrifuged at 1600 xg, 4°C for 15 min twice to clear off the cells and cell debris. EVs were pelleted from the supernatant by ultracentrifugation at 100,000 xg, for 70 min at 4°C using a micro‐ultracentrifuge (Hitachi CS 150NX) and washed once with PBS, then resuspended in 50 μl PBS.

#### 4.2.3 Nanoparticle tracking analysis

We analysed the concentration and size distribution of EVs using a NanoSight NS300 nanoparticle tracking analyser (Malvern, UK) or ZetaView nanoparticle tracking analyser (Particle Metrix, Germany). We loaded a total of 0.5 ml of EVs into the sample chamber, recorded three videos for each sample, and analysed the recordings with similar detection thresholds within each batch according to the recommended protocols (Waldman et al., 2020). We used the average concentration from three recordings as the EV concentration.

#### 4.2.4 Protein concentration analysis

We used bicinchoninic acid (BCA) assay to measure the protein concentrations with a Pierce BCA Protein Assay Kit (Thermo Fisher, USA). Standards were generated with BSA (New England Biolabs, USA) using serial dilution. A total of 25 μl of either samples or standards were loaded into each well of a 96‐well plate and 250 μl of BCA working reagent was added. The plate was put on a plate shaker for 30 s for a thoroughly mixing and then incubated at 37°C for 30 min. We cooled the plate to room temperature (RT) and then measured the absorbance at 562 nm using the Synergy H1 microplate reader (Biotek, USA).

#### 4.2.5 Transmission electron microscopy (TEM)

As previously described (Vu et al., 2019), we fixed the EVs using 4% paraformaldehyde, loaded them onto a copper grid and performed subsequent washes using PBS, 1% glutaraldehyde, and distilled water. We then incubated the EVs with 4% uranyl acetate and imaged the EVs using a FEI Tecnai 12 BioTwin TEM (FEI/Phillips, USA) at 100 kV.

#### 4.2.6 Magnetic bead‐based flow cytometry for EV surface protein analysis

We incubated human EVs of similar counts (∼ 5.0×10^10^ particles) with anti‐human CD63 magnetic beads (∼30 μg, Thermo Fisher, USA) with orbital rotation and vibration mixing on a HulaMixer (Thermo Fisher, USA) for 20 h at 4°C. EV‐bead complexes were isolated using a DynaMag magnet (Thermo Fisher, USA), washed with 0.22‐μm‐filtered 0.1% BSA‐supplemented PBS three times. To compare the EV binding, we collected the supernatant from the first wash and measured concentration of unbound EVs using ZetaView nanoparticle tracking analysis instrument (Particle Metrix, Germany). For further analysis, bead‐bound EVs were stained with fluorophore‐conjugated anti‐human CD63, anti‐human ITGAV, anti‐human ITGB1, anti‐human ITGB4, anti‐human ITGB5, and anti‐human CD11b antibodies (BioLegend, USA) for 45 min at RT. Stained EV‐bead complexes were washed three times with 0.22‐μm‐filtered 0.1% BSA‐supplemented PBS before being analysed using a CytoflexS flow cytometer (Beckman Coulter, USA). Mouse EVs were incubated with in‐house conjugated anti‐mouse CD63 magnetic beads, washed, stained with fluorophore‐conjugated anti‐mouse CD63, anti‐mouse ITGAV, anti‐mouse ITGB1, and anti‐mouse CD11b antibodies (BioLegend, USA), and analysed in a similar fashion. We generated in‐house conjugated anti‐mouse CD63 magnetic beads by incubating streptavidin‐conjugated magnetic beads (Thermo Fisher, USA) with biotin‐labelled anti‐mouse CD63 antibody (Miltenyi Biotec, Germany) with orbital rotation and vibration mixing on a HulaMixer (Thermo Fisher, USA) for 45 min at RT. We generated anti‐human ITGAV magnetic beads in a similar manner by incubating streptavidin‐conjugated magnetic beads (Thermo Fisher, USA) with biotin‐labelled anti‐human ITGAV antibody (BioLegend, USA). Three washes were performed using 0.22‐μm‐filtered 0.1% BSA‐supplemented PBS after the conjugation. Fluorescence was analysed using a CytoFLEX S flow cytometer (Beckman). The percentage of beads with positive integrin signal reflected the integrin levels as we used equal counts of EVs and beads across assays, and the beads were saturated at the concentration we used.

### 4.3 ELISA assay for ITGAV quantification

50 μl EVs isolated from patients' whole blood samples were lysed in 0.1% Triton‐X to the final volume of 100 μl. The level of ITGAV was assessed using the ITGAV ELISA kit (Aviva Systems Biology, USA) according to the manufacturer's instruction. Absorbance at 450 nm was detected using the Tecan Spark 10M multimode microplate reader.

### 4.4 Mass spectrometry

#### 4.4.1 In‐gel trypsin digestion of SDS gel bands

The protein bands for a pair of samples from an SDS‐PAGE gel were cut into ∼1 mm cubes and subjected to in‐gel digestion followed by extraction of the tryptic peptide, as reported previously (Vestad et al., 2017). The excised gel pieces were washed consecutively in 200 μl distilled water, 100 mM ammonium bicarbonate (Ambic)/acetonitrile (1:1) and acetonitrile. The gel pieces were reduced with 70 μl of 10 mM DTT in 100 mM Ambic for 1 h at 56°C, alkylated with 100 μl of 55 mM Iodoacetamide in 100 mM Ambic at RT in the dark for 60 min. After wash steps as described above, the gel slices were dried and rehydrated with 50 μl trypsin in 50 mM Ambic, 10% ACN (20 ng/μl) for 16 h at 37°C. The digested peptides were extracted twice with 70 μl of 50% acetonitrile, 5% formic acid (FA) and once with 70 μl of 90% acetonitrile, 5% FA. Extracts from each sample were combined, filtered by a 0.22‐μm spinning unit, and lyophilized.

#### 4.4.2 Protein Identification by nano LC/MS/MS Analysis

The in‐gel tryptic digests were reconstituted in 20 μl of 0.5% FA for nanoLC‐ESI‐MS/MS analysis, which was carried out using an Orbitrap Fusion Tribrid mass spectrometer (Thermo Fisher, USA) equipped with a nanospray Flex Ion Source, and coupled with a Dionex UltiMate3000RSLCnano system (Thermo Fisher, USA) (Thomas et al., 2017, Yang et al., 2007). The gel extracted peptide samples (20 μl) were injected onto a PepMap C‐18 RP nano trapping column (5 μm, 100 μm i.d x 20 mm) at 20 μl/min flow rate for rapid sample loading and then separated on a PepMap C‐18 RP nano column (2 μm, 75 μm x 25 cm) at 35°C. The tryptic peptides were eluted in a 120 min gradient of 5% to 38% acetonitrile (ACN) in 0.1% FA at 300 nl/min, followed by a 7 min ramping to 90% ACN‐0.1% FA and an 8 min hold at 90% ACN‐0.1% FA. The column was re‐equilibrated with 0.1% FA for 25 min prior to the next run. The Orbitrap Fusion is operated in positive ion mode with spray voltage set at 1.6 kV and source temperature at 275°C. External calibration for FT, IT and quadrupole mass analysers was performed. In data‐dependent acquisition (DDA) analysis, the instrument was operated using FT mass analyser in MS scan to select precursor ions followed by 3 s “Top Speed” data‐dependent CID ion trap MS/MS scans at 1.6 m/z quadrupole isolation for precursor peptides with multiple charged ions above a threshold ion count of 10,000 and normalized collision energy of 30%. MS survey scans at a resolving power of 120,000 (fwhm at m/z 200), for the mass range of m/z 375–1575. Dynamic exclusion parameters were set at 40 s of exclusion duration with ±10 ppm exclusion mass width. All data were acquired under Xcalibur 4.3 operation software (Thermo Fisher, USA).

#### 4.4.3 Data analysis

The DDA raw files for CID MS/MS were subjected to database searches using Proteome Discoverer (PD) 2.3 software (Thermo Fisher, USA) with the Sequest HT algorithm. Processing workflow for precursor‐based quantification. The PD 2.3 processing workflow containing an additional node of Minora Feature Detector for precursor ion‐based quantification was used for protein identification and protein relatively quantitation analysis between samples. The database search was conducted against a *Homo sapiens* database containing 20,367 sequences downloaded from UniProt, or a *Mus musculus* database that has 47,929 sequences downloaded from NCBInr plus a common contaminant (246 entries) database. Two‐missed trypsin cleavage sites were allowed. The peptide precursor tolerance was set to 10 ppm and fragment ion tolerance was set to 0.6 Da. Variable modification of methionine oxidation, deamidation of asparagines/glutamine and fixed modification of cysteine carbamidomethylation, were set for the database search. Identified peptides were further filtered for a maximum 1% false discovery rate (FDR) using the Percolator algorithm in PD 2.3 along with additional peptide confidence set to high and peptide mass accuracy ≤5 ppm. The final protein IDs contained protein groups that were filtered with at least two peptides per protein. Relative quantitation of identified proteins between the two samples was determined by the Label Free Quantitation (LFQ) workflow in PD 2.3. The precursor abundance intensity for each peptide identified by MS/MS in each sample was automatically determined and their unique plus razor peptides for each protein in each sample were summed and used for calculating the protein abundance by PD 2.3 software without normalization. Protein ratios were calculated based on pairwise ratio for paired samples. Human and mouse EV protein entries were obtained from Vesiclepedia (Yang et al., 2018) and Venn diagrams were generated using Bioinformatics & Evolutionary Genomics tools (Ghent University, Belgium). Gene ontology analysis and pathway analysis were facilitated using a database for annotation, visualization and integrated discovery version 6.8 (Kalra et al., 2012) and Kyoto Encyclopedia of Genes and Genomes (Huang et al., 2008). Protein‐protein interaction network analysis was performed using STRING databases (Kanehisa et al., 2021) and visualized using Cytoscape and BioRender.

### 4.5 Animal experiments

All mouse experiments were performed in accordance with study protocols approved by the institutional animal ethics committee. NSG mice (NOD.Cg‐Prkdc^scid^ Il2rg ^tm1Wjl ^/SzJ), NSG‐SGM3 mice (NOD.Cg‐ Prkdc^scid^ Il2rg ^tm1Wjl^ Tg(CMV‐IL3,CSF2,KITLG)1Eav/MloySzJ) and nude mice (Nu/J) were purchased from the Jackson Laboratory (USA) and bred in our facility.

#### 4.5.1 In vivo metastasis models

5×10^5^ CA1a‐luc or MB‐231‐luc cells were injected per 6‐week‐old female nude mice intravenously via the tail vein. Bioluminescent images of the mice were acquired every week using the IVIS Lumina III system 10 min after an i.p. injection of D‐luciferin at 150 mg/kg (Caliper Life Sciences), under anaesthesia with 1% isoflurane.

Similarly, female NSG‐SGM3 mice at 6–8 weeks old were injected with 1×10^6^ CA1a cells expressing either scrambled shRNA or *Gal3* shRNA in the tail vein. After 9 weeks, the mice were sacrificed, and the lung tissue was collected.

#### 4.5.2 ITGAV detection in circulating EVs from xenografted mice

A total of 250,000 CA1a or MB‐231 cells were resuspended in PBS‐Matrigel (Corning, USA) solution (1:1 ratio) and implanted in the flanks of 8‐week‐old female NSG mice. When tumours reached 15 mm in diameter, the mice were sacrificed, and the whole blood was taken collected. The blood was allowed to clot undisturbed for 30 min at RT and then centrifuged at 1500 × g for 15 min at 4°C. We collected the serum from the resulting supernatant and pooled the serum from two mice of the same group. Circulating EVs were prepared from equal volumes of serum inputs using ExoQuick Ultra EV Isolation Kit for Serum (System Biosciences, USA), following the manufacturer's protocol. We then incubated the isolated circulating EVs with anti‐human CD63 magnetic beads (Thermo Fisher, USA), and stained them with fluorophore‐conjugated anti‐human ITGAV antibody (BioLegend, USA) for flow cytometry analysis, as described above.

#### 4.5.3 Immunofluorescence staining of tumour sections

As previously described (Vu et al., 2019), CA1a or MB‐231 xenografted tumours were fixed in formalin and then embedded in paraffin. We prepared sections of 5 μm and dewaxed them in xylene. Rehydration was performed in down‐gradient alcohols (100%, 95%, 70%) and later water. We conducted the antigen retrieval by superheating the sections in a laboratory microwave oven for a further 30 min after the pre‐boiling of Envision Flex Target Retrieval high pH Solution (Dako, Denmark). Sections were allowed at least 20 min to cool down in the antigen retrieval solution. Rabbit anti‐ITGAV primary antibody (Abcam, dilution 1:500) was applied to sections and incubated for 30 min at RT after blocking. Signal detection was facilitated by adding goat anti‐rabbit secondary antibody conjugated with HRP (Perkin Elmer, USA), followed by Opal 570 fluorophore (Perkin Elmer, USA), with incubation for 10 min at RT each. Sections were then re‐microwaved with a new retrieval solution to strip unbound anti‐ITGAV antibody and to unmask CD63 antigens. Blocking reagent was re‐applied for 10 min at RT. After incubating with mouse anti‐CD63 antibody (Abcam, dilution 1:500) for 30 min at room temperature, we added goat anti‐mouse secondary antibody conjugated with HRP (Perkin Elmer, USA), followed by Opal 480 fluorophore (Perkin Elmer, USA), with incubation for 10 min at RT each. We acquired the images using an LSM‐880 NLO confocal laser scanning microscope (Zeiss, Germany) and presented them with pseudo‐colours. Images of five representative fields were captured per tumour section and we calculated and used the average coefficient from five images per tumour.

#### 4.5.4 Haematoxylin and eosin staining for metastasis quantification

The lungs were excised from NSG‐SGM3 mice and fixed in 10% formalin (Sigma‐Aldrich, USA) for 48 h. The tissue was dehydrated sequentially in one bath of 70% ethanol, one bath of 80% ethanol, one bath of 90% ethanol and three baths of 100% alcohol. The tissues were cleared in three baths of Histo‐Clear (National Diagnostics, USA), soaked in two baths of paraffin wax at 65°C and embedded in paraffin. Paraffin blocks were cut at 4 μm using a microtome (Leica RM2255) and dried overnight at RT. Sections were deparaffinised in Histo‐Clear for 5 min twice, then immersed into two baths of 100% ethanol, one bath of 90% ethanol, one bath of 70% ethanol and one bath of deionized water, each for 2 min. After washing with water, sections were stained with Harris's haematoxylin for 7 min and differentiated in freshly made 70% alcohol with 0.1% HCl for 30 sec. Stained sections were washed and blued under running tap water for 5 min or until the excess stain was washed away. Sections were then counterstained with Eosin Y ethanol solution (Sigma‐Aldrich, USA) for 10 sec. After a quick wash three times in water, sections were dehydrated in one bath of 70% ethanol, one bath of 90% ethanol and two baths of 100% ethanol, each for 30 sec. Sections were then cleared with Histo‐Clear for 10 min, dehydrated in 100% ethanol for 7 min, then dried overnight and mounted with mounting medium and coverslip. Images of H & E stained lung sections were captured using the Leica DM6B Upright Microscopes system and analysed using ImageJ software in a blinded manner (version 1.53).

#### 4.5.5 Mouse primary fibroblast isolation

Mouse primary fibroblast were isolated from female NSG‐SGM3 mice as previously described (Vu et al., 2019). Briefly, the mice were euthanized, the ears were removed and sterilized with 70% ethanol for at least 5 min. The ears were then cut into small pieces and incubated with RPMI supplemented with 10% FBS, 1× penicillin‐streptomycin, 1× plasmocin (1:1) and 5 mg/ml collagenase type IV (Thermo Fisher Scientific, USA) at 37°C, with shaking at 250 rpm for 90 min. Tissue suspension was filtered through 70 μm strainer, washed once with 10 ml of RPMI with 10% FBS at 700 xg for 5 min. Cell pellets were resuspended in 10 ml RPMI with 10% FBS and seeded into 100 mm plate. After 3 days, the medium was replaced and the cells were monitored daily until they reached 80% confluence (passage 1). Fibroblasts were subcultured and used within the 1^st^ to 5^th^ passage.

### 4.6 Immunohistochemistry staining of tumour sections from breast cancer patients

We collected formalin‐fixed, paraffin‐embedded (FFPE) tumour samples of breast cancer from 51 breast cancer patients (including seven cases of luminal A, 29 cases of luminal B, seven cases of HER2‐enriched and eight cases of basal‐like subtypes) who were hospitalized for surgery at the First Affiliated Hospital of the Guangxi Medical University, China. Study inclusion criteria included: (1) the patients were women; (2) the diagnosis of primary breast cancer was confirmed histopathologically; (3) the patients were diagnosed for the first time without receiving breast cancer treatments or surgery before; and (4) medical records were available and patients agreed to grant access for the purpose of retrieving clinicopathological parameters. Study exclusion criteria included: (1) the patients were men; (2) the patients received prior therapy or surgery to treat breast cancer; and (3) the patients were clinically immunocompromised or were experiencing major systemic diseases. Clinical data were de‐identified to protect the privacy of the study participants. We prepared FFPE sections of 5 μm, followed by deparaffinization and rehydration in xylene, down‐gradient alcohols (100%, 95%, 85%, 75%, 50%) and water, heat‐mediated antigen retrieval, and peroxidase blocking. Rabbit anti‐ITGAV antibody (Abcam, dilution 1:500) was then applied to sections and incubated for 60 min at RT. Detection was facilitated by Supervision Mouse/Rabbit‐HRP Broad Spectrum Detection System (Long Island Biotech, member of Mindray Medical, China), according to the manufacturer's protocols. Haematoxylin was applied for nuclear counterstain. We scored the membrane and cytoplasm staining of tumour cells accordingly and the final score for each image was the weighted average of IHC scores multiplied by percentages of tumour cells within each scale. We captured images of five representative fields for each tumour section and the average score from five images was considered as the final score for each case. The study protocol was approved by the institutional review board, and written informed consents were obtained from all study participants. Histopathological diagnosis was reviewed, and images were examined by two board‐certified pathologists (GC and JYL) independently. Cancer staging was referred to the American Joint Committee on Cancer (AJCC) Cancer Staging Manual, Eighth Edition (Szklarczyk et al., 2019).

To better understand the prognostic values of integrin αv and CD63 co‐expression, we studied the association of the co‐expression with a set of clinicopathological parameters in patients with breast carcinoma. A total of 128 patients with breast carcinoma were selected from a set of inclusion and exclusion criteria. The inclusion criteria are all the breast carcinoma cases in the National University Hospital from year 2012 to 2014 with available clinical and histological data, and sufficient FFPE cancer tissue. Exclusion criteria include cases that show poor tissue condition, not suitable for immunohistochemical staining. Clinical data collected include gender, age, laterality of tumour, type of surgery, presence of neoadjuvant treatment, initial stage pre neoadjuvant, clinical and radiological presence of recurrence, and presence of distant metastasis. The histological data collected include the histological type as according to WHO Classification of Breast Tumours (5^th^ edition, 2019) (Edge & Edge, 2017), histological grading as according to the Nottingham Histological Score, biologic markers status (estrogen receptor (ER), progesterone receptor (PR), and human epidermal growth factor receptor 2 (HER2) immunoreaction), presence, extent and grade of in‐situ carcinoma component, presence of lymphovascular invasion (LVI), and status of margin. The cases are staged according to the Breast Cancer Staging Manual by American Joint Committee on Cancer (8^th^ Edition, 2017) (Editorial Board 2019). In addition, we obtained the normal breast tissue adjacent to the tumours in eight anonymized patients with breast cancer. We found that four samples had only fat tissues and found four samples contain normal mammary glands. Hence, we used the four samples with normal mammary glands for the staining. The study protocol was approved by the Domain Specific Review Board (DSRB) committee of the National University Hospital (Singapore).

Tissue microarray was prepared from each of the cases’ FFPE tissue blocks (Table 5). IHC staining was performed using antibodies against CD63 (Santa Cruz, Cat no. sc‐15363) and CD51 (Abcam, Cat no. ab179475). The IHC staining pattern was assessed by two certified pathologists and scored as “1” (negative) if only red cytoplasmic staining (CD51) or only brown granular cytoplasmic staining (CD63), or very weak/negligible/no staining for both were observed; and scored as “2” (positive) if both red (CD51) and brown granular cytoplasmic (CD63) were observed. Cases were pooled into two separate groups in which the first group consisted of early‐stage non‐invasive/non‐metastatic breast carcinoma (<T3, N0, M0, no LVI) and the second group consisted of locally advanced breast carcinoma (T3 & above), cases with lymph node invasion (lymph node infiltrating tumour cells‐ITC, N1 & above), metastatic (M1) and/or lymphovascular invasion (LVI); with reference to the MD Anderson definition. The Chi‐square test was used to test the associations between co‐expression of CD63 and CD51 with disease characteristics.

### 4.7 In vitro assays

#### 4.7.1 Quantitative reverse transcription PCR

TRIzol (Thermo Fisher, USA) was used for RNA extraction, according to the manufacturer's protocol. We diluted the stock RNA to 50 ng/μl for reverse transcription. A total of 500 ng total RNA was applied for reverse transcription of mRNA, with a High‐Capacity cDNA Reverse Transcription Kit (Thermo Fisher, USA). Quantitative PCR (qPCR) was performed using a Ssofast Green qPCR kit (BioRad, USA) for mRNA quantification in a CFX‐96 qPCR machine (BioRad, USA) or QuantStudio 6 Flex Real Time PCR system (Applied Biosystems, USA). mRNA levels were normalized to *GAPDH* or *ATCB* mRNA levels. Primer design was facilitated using PrimerBank (Amin et al., 2017) (10.1093/nar/gkr1013) and Primer‐BLAST (Wang et al., 2011). We attached the primer sequences in Table S6.

#### 4.7.2 Western blots

Protein concentration was determined using the BCA assay, as described above. As described earlier (Vu et al., 2019), we diluted protein samples in reducing buffer (4x Laemmli sample buffer (Biorad, USA) with 10% β‐mercaptoethanol), and heated the mixture for 10 min at 95°C. For non‐reducing gel electrophoresis, the samples were diluted in 4x Laemmli Sample Buffer without β‐mercaptoethanol. Similar amounts of protein (35 μg of total cell lysate) were loaded onto a 10% SDS‐PAGE resolving gel with a 4% stacking gel (for galectin‐1, a 15% SDS‐PAGE resolving gel with a 6% stacking gel). Electrophoresis was conducted at 70 V for stacking and 120 V for resolving till the sample dye reached the gel bottom. We used wet transfer to blot the samples to a PVDF membrane activated by 100% methanol. The membrane after transfer was blocked with 5% skimmed milk (BD Biosciences, USA) in TBST buffer (20 mM Tris base, 150 mM NaCl and 0.1% Tween 20) for 1 h at RT. The membrane was then incubated with primary antibodies, anti‐TSG101 antibody (Santa Cruz), anti‐ALIX antibody (Santa Cruz), anti‐Calnexin (Santa Cruz), anti‐Gal‐1 antibody (Cell Signalling Tech, USA), anti‐Gal‐3 antibody (Abcam, UK) and anti‐GAPDH antibody (Abbkine, USA) in TBST containing 5% skimmed milk overnight at 4°C and washed three times (5 min each time) with TBST at RT on a plate shaker. We then incubated the membrane with peroxidase‐labelled anti‐mouse or anti‐rabbit secondary antibody (Vector Laboratories, UK) in TBST with 5% skimmed milk for 1 h at RT. Membrane was then washed three times (5 min each time) with TBST at RT on a plate shaker before signal developing with the WesternBright ECL HRP substrate kit (Advansta, USA) using a ChemiDoc Touch Imaging System (BioRad, USA).

#### 4.7.3 Immunofluorescence staining of cells

Cover slips were washed with ethanol, coated with poly‐D‐lysine (Millipore, USA) for 1 h at RT and then washed with 0.22‐μm‐filtered PBS. A total of 200,000 cells were seeded onto the coated cover slip in each well of a 12‐well plate overnight. Next, cells were washed with PBS three times and fixed in 4% paraformaldehyde for 15 min at RT. Cells were treated with 5% BSA (Sigma Aldrich, USA) with 0.2% triton X‐100 (Sigma Aldrich, USA) for 60 min at RT and incubated with anti‐CD63 antibody (Abcam, UK) and anti‐ITGAV (Abcam, UK) or anti‐ITGB1 (eBioscience, USA) antibodies in with 3% BSA (Sigma Aldrich, USA) with 0.05% triton X‐100 (Sigma Aldrich, USA) overnight at 4°C. Cells were washed three times with PBS containing 0.05% Triton before incubation with anti‐mouse‐AF488 and anti‐rabbit‐647 antibodies (Jackson ImmunoResearch, USA) for 60 min at RT on a plate shaker, and then washed three times with PBS. Cells were incubated with Hoechst Stain (Sigma Aldrich, USA) in PBS for 15 min at RT and washed three times with PBS. The cover slips were mounted on glass slides with ProLong Glass Antifade Mountant (Thermo Fisher, USA). Images were acquired using a Zeiss LSM 880 Confocal Microscope.

#### 4.7.4 Fibronectin binding assay of cells

Untreated, black 96‐well plates were coated with human fibronectin (0.3 μg/ml, 100uL) overnight at 4°C and then blocked with 1% BSA for 1h at RT, followed by three washes. A total of 100,000 CA1a cells were resuspended in DMEM with addition of compound 8 (C8) (Chemscene, USA) to reach desired concentrations, and incubated for 30 min at 4°C. Cells were then dispensed into wells of fibronectin‐coated black 96‐well plates and incubated for 30 min at RT with a 5% CO_2_ supply. Calcein AM was added to the wells to reach the final concentration of 2 μM dye per 100,000 cells, followed by another incubation for 30 min at RT with 5% CO_2_ supply and two gentle washes with PBS to remove unbound cells. We read fluorescence at 494/517 nm using a microplate reader after adding 100 μl PBS to each well.

#### 4.7.5 Fibronectin binding assay of EVs

Untreated, black 96‐well plates were coated with human fibronectin at a concentration of 1.28 μg/ml, 100 μl per well overnight at 4°C. BSA 1 mg/ml was used to coat the wells designated for negative control overnight at 4°C. EVs from CA1a‐luc cells were incubated with C8 (Chemscene, USA) at the desired concentrations for 30 min at 4°C, dispensed into the wells, and put on a plate shaker for 2 h at RT. We then washed the wells three times with PBS, added 100 μl coelenterazine native at 2 μg/ml (Nanolight, USA) into each well, and read bioluminescence signals using Tecan Spark multimode microplate reader (Tecan, Switzerland).

#### 4.7.6 Fibroblast activation assay

EV‐depleted RPMI medium supplemented with 10% FBS was prepared in a similar manner to EV‐depleted DMEM medium, as described above. 48‐well plates were coated with human fibronectin at concentration of 5 μg/ml, 200 μl per well overnight at 4°C. The plate was washed twice with sterile PBS. Purified EVs from CA1a cells with or without scrambled or Gal‐3 shRNA were added into the plate at 5×10^9^ EVs/well in PBS, and the plate was placed on a shaker at a speed of 300 rpm for 2 h at RT. The plate was gently washed twice with sterile PBS. Mouse primary fibroblasts were seeded into EV‐pretreated wells at the density of 50,000 cell per well in EV‐depleted RPMI complete medium and incubated at 37°C, 5% CO_2_. Fibroblasts were starved in serum‐free media for 24 h before treatment. After 72 h, the plate was gently washed twice with PBS, and 0.5 ml of Trizol was added for RNA extraction and RT‐qPCR.

### 4.8 Statistical analysis

We used GraphPad Prism 7 to perform all the statistical analysis using Student's one‐tailed t‐tests, unless stated otherwise. A p‐value less than 0.05 was considered significant, based on at least three independent replicates. Additionally, clinical data were further analysed using Stata 16 and IBM SPSS Statistics 26.0, Mann Whitney's test and Chi‐square test.

## CONFLICT OF INTEREST

Minh T. N. Le is a scientific co‐founder and advisor of Carmine Therapeutics, a company that develops extracellular‐vesicle‐based therapies. Other authors declare no conflict of interest.

## GEOLOCATION INFORMATION

The research was performed in Singapore, USA, Hong Kong, and China.

## Supporting information

Supporting Information

Supporting Information

Supporting Information

Supporting Information

Supporting Information

Supporting Information

Supporting Information

## Data Availability

Proteomic data are available in the PRIDE database (PXD025616). Other raw data are available upon request.
