# Supplementary material for: αvβ1 integrin is enriched in extracellular vesicles of metastatic breast cancer cells: A mechanism mediated by galectin‐3
Source: J Extracell Vesicles. 2022 Aug 3;11(8):e12234. doi: 10.1002/jev2.12234 (PMC9451529; doi:10.1002/jev2.12234)
Supplement: Supplementary file 7 — Supporting Information [file JEV2-11-e12234-s003.pdf]

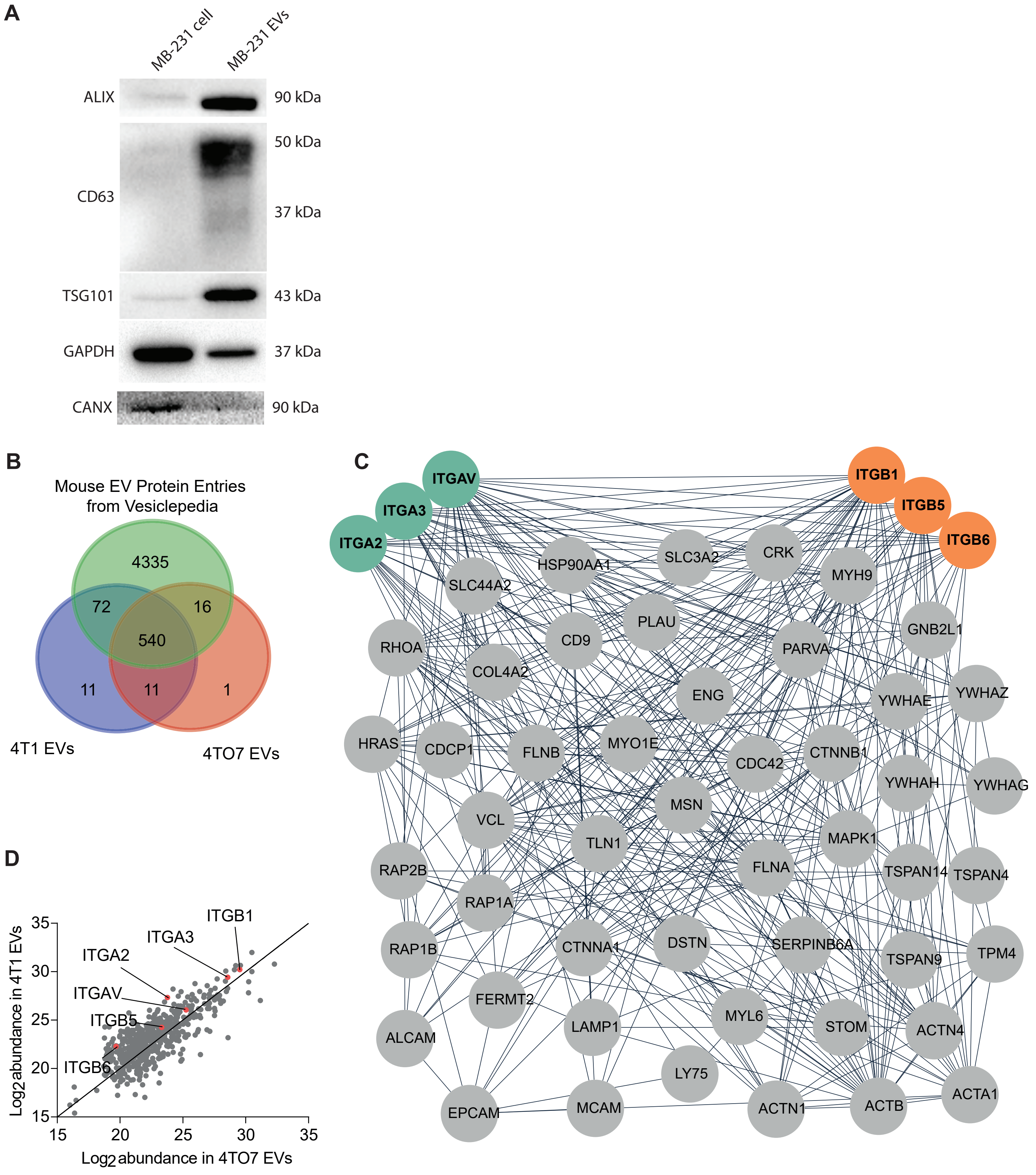

Supplementary Figure 1. Proteomic profiling of mouse breast cancer cell-derived EVs.
(A) Western blot analysis of proteins in MB-231 cells and EVs. **(B)** Venn diagram showing overlaps of identified mouse EV proteins from proteomic profiling with known mouse EV protein entries from Vesiclepedia. **(C)** Scatter plot representing differentially expressed proteins identified in 4T1 and 4TO7 EVs. **(D)** Protein-protein interaction network analysis of proteins enriched in 4T1 EVs compared to 4TO7 EVs.

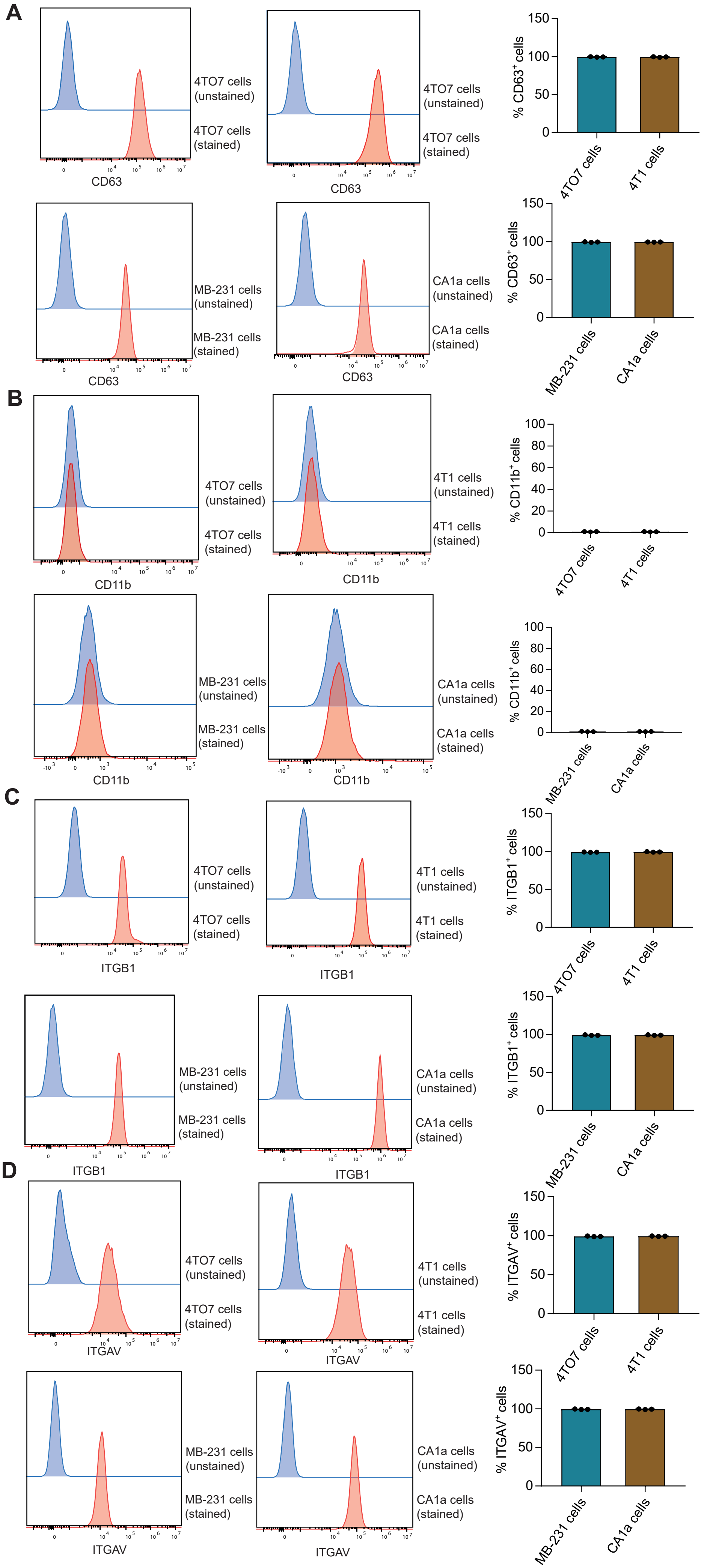

Supplementary Figure 2

Flow cytometry analysis of cell surface markers in breast cancer cells.
Flow cytometry histograms and graphs of (A) positive control CD63, (B) negative control CD11b, (C) ITGB1, and (D) ITGAV in 4TO7, 4T1, CA1a, and MB-231 cells.

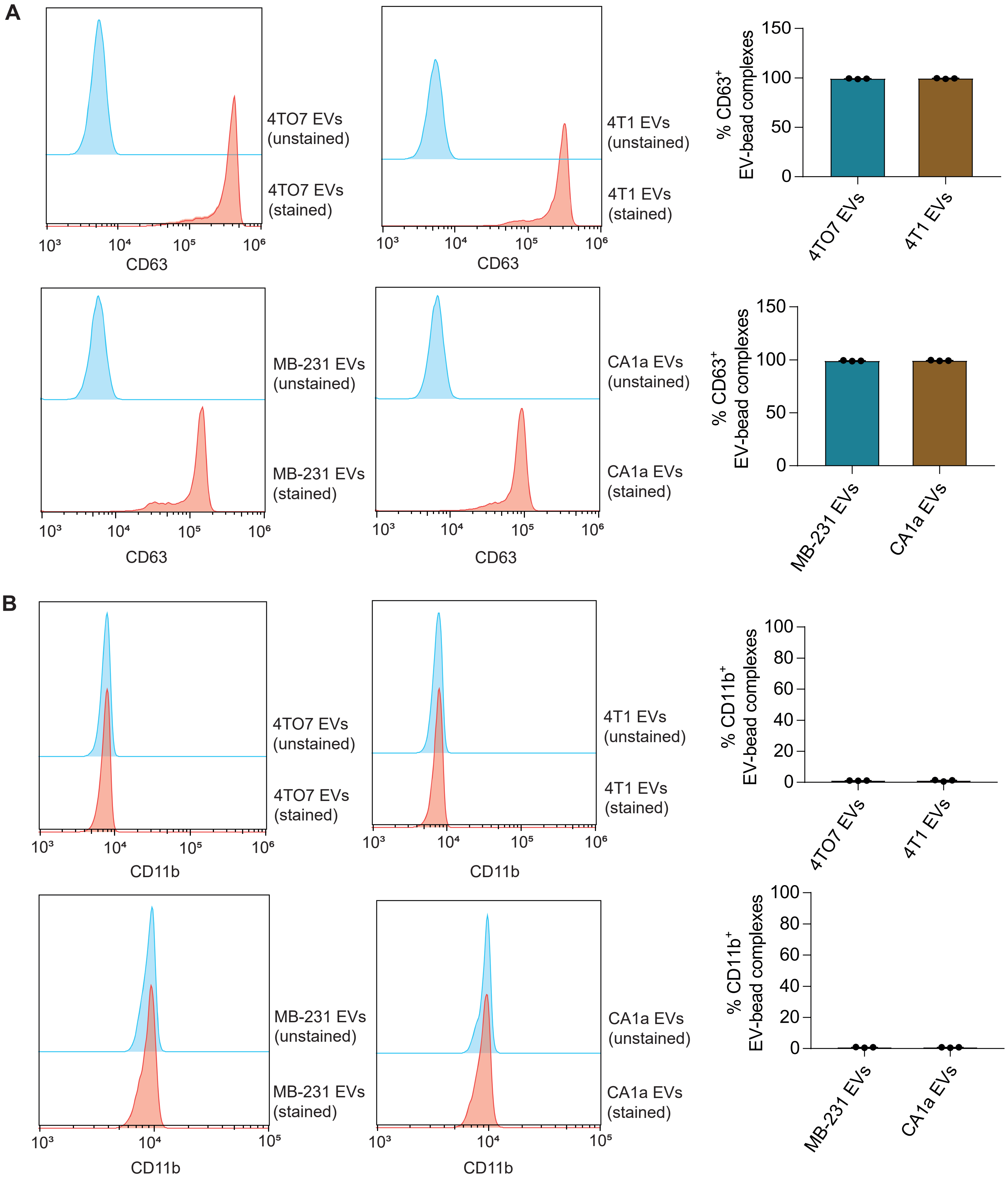

Supplementary Figure 3. Flow cytometry analysis of CD63 and CD11b on the surface of EVs from breast cancer cells. Flow cytometry histograms and graphs of **(A)** positive control CD63, and **(B)** negative control CD11b on bead-captured 4TO7, 4T1, CA1a, and MB-231 EVs.

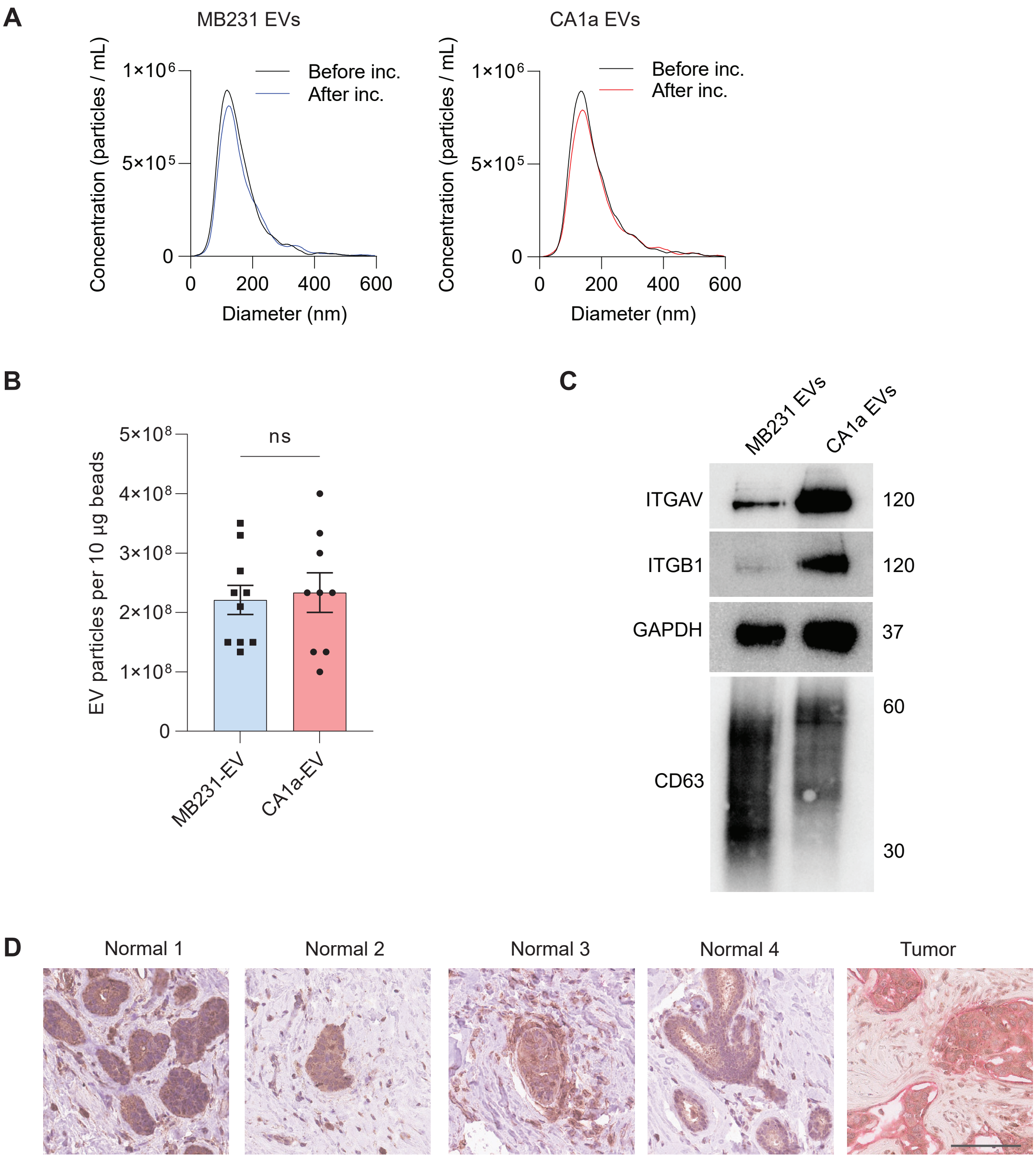

Supplementary Figure 4. Quantification of EVs on beads and EV protein comparison. (A) Nanoparticle tracking analysis of EVs before and after incubation (inc.) with magnetic beads coated with anti-CD63 antibody, determined using a ZetaView® nanoparticle tracking analyzer. **(B)** Number of bead-bound EV particles, equal to the number of input EVs less the number of unbound EVs in the supernatant. N = 9 including 3 biological replicates each of 3 technical replicates. ns, non-significant, determined using Student's t-test. **(C)** Western blot analysis of EV proteins separated using a non-reducing gel. Numbers on the right indicate molecular weight (kDa). **(D)** Immunostaining of CD63 (brown) and ITGAV (red) in normal breast tissues and tumor samples of breast cancer patients. Scale bar, 100 micrometers.

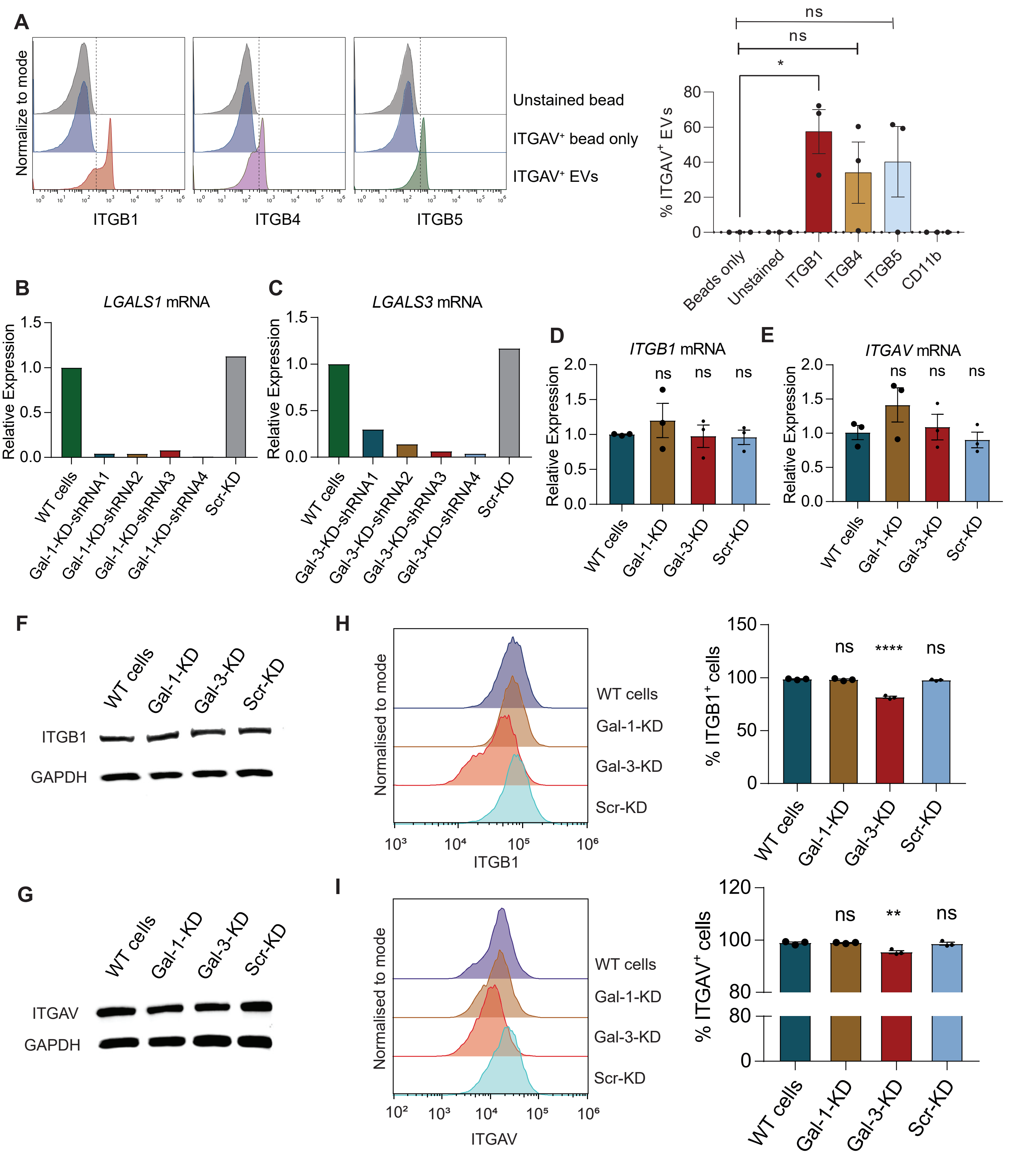

Supplementary Figure 5. shRNA-mediated knockdown of *Gal-1* and *Gal-3* in CA1a cells.
(A) Flow cytometry analysis of ITGB1, ITGB4, and ITGB5 on ITGAV+ EVs from CA1a cells, captured by anti-human ITGAV beads. **(B, C)** shRNA screening efficiency of Gal-1 and Gal-3 using qPCR. **(D-F)** Knockdown of either *Gal-1* or *Gal-3* did not change mRNA (D-E) or protein levels (F-G) of ITGB1 and ITGAV. **(H-I)** Flow cytometry analysis showing the effects on the cell surface ITGB1 and ITGAV after the knockdown of *Gal-3* but not *Gal-1*.
